# Integrated analysis of glycan and RNA in single cells

**DOI:** 10.1016/j.isci.2021.102882

**Published:** 2021-07-17

**Authors:** Fumi Minoshima, Haruka Ozaki, Haruki Odaka, Hiroaki Tateno

**Affiliations:** 1 Cellular and Molecular Biotechnology Research Institute, National Institute of Advanced Industrial Science and Technology (AIST), Tsukuba Central 6, 1-1-1 Higashi, Tsukuba, Ibaraki 305-8566, Japan; 2 Bioinformatics Laboratory, Faculty of Medicine, University of Tsukuba, 1-1-1 Tennodai, Tsukuba, Ibaraki 305-8575, Japan; 3 Center for Artificial Intelligence Research, University of Tsukuba, 1-1-1 Tennodai, Tsukuba, Ibaraki 305-8577, Japan; 4 JST PRESTO, Tsukuba Central 6, 1-1-1 Higashi, Tsukuba, Ibaraki 305-8566, Japan

**Keywords:** Molecular biology, Molecular Structure, Molecular biology experimental approach, Cell biology, Organizational aspects of cell biology

## Abstract

Single-cell sequencing has emerged as an indispensable technology to dissect cellular heterogeneity but never been applied to the simultaneous analysis of glycan and RNA. Using oligonucleotide-labeled lectins, we first established lectin-based glycan profiling of single cells by sequencing (scGlycan-seq). We then combined the scGlycan-seq with single-cell transcriptome profiling for joint analysis of glycan and RNA in single cells (scGR-seq). Using scGR-seq, we analyzed the two modalities in human induced pluripotent stem cells (hiPSCs) before and after differentiation into neural progenitor cells at the single-cell resolution. The combination of RNA and glycan separated the two cell types clearer than either one of them. Furthermore, integrative analysis of glycan and RNA modalities in single cells found known and unknown lectins that were specific to hiPSCs and coordinated with neural differentiation. Taken together, we demonstrate that scGR-seq can reveal the cellular heterogeneity and biological roles of glycans across multicellular systems.

## Highlights

• scGlycan-seq was developed for single-cell glycan profiling by sequencing
• scGR-seq was developed for joint analysis of glycan and RNA in single cells
• scGR-seq separated the two distinct cell types clearer than either one of them
• scGR-seq identified lectins related to pluripotency and neural differentiation

## Graphical abstract

**Figure:**
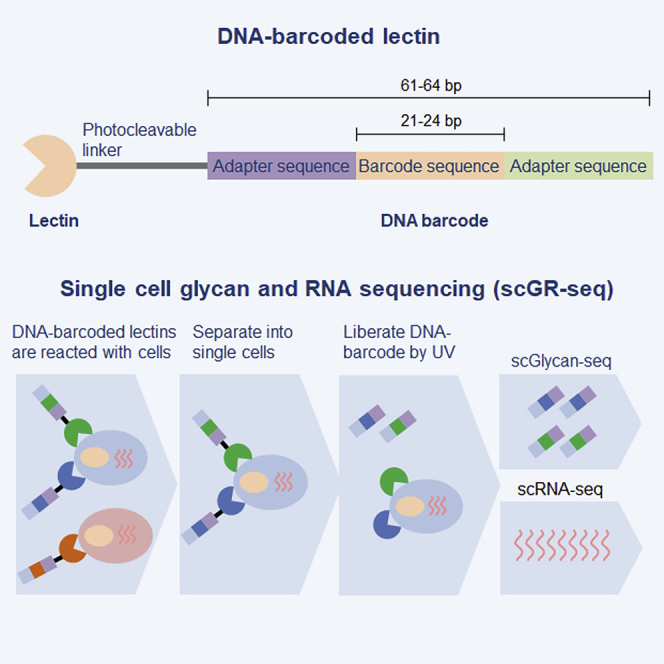

## Introduction

Glycans are the most structurally diverse and rapidly evolving major class of molecules, which present at the surface of all living cells and play crucial roles in diverse biological processes (Varki, 2017). Glycan structures have been known to vary depending on cell types and states. Therefore, cell surface glycans are often referred to as “cell signature” that reflect cellular characteristics. Indeed, most of the stem cell markers (Kannagi et al., 1983; Kawabe et al., 2013; Schopperle and DeWolf, 2007; Tang et al., 2011; Wu et al., 2019) and serum tumor markers are glycoconjugates (Munkley, 2019; Pearce, 2018; Varki et al., 2015). Glycans are the secondary products of genes synthesized by the orchestration of many proteins, such as glycosyltransferases and glycosidases. Despite advances in technology, there is no established method to predict the precise glycan structures only from the gene expression profiles. Therefore, it is vital to develop methods to analyze cell surface glycans directly. In this sense, different strategies have been undertaken to analyze the glycome, including mass spectrometry (MS), high-performance liquid chromatography (HPLC), nuclear magnetic resonance, and capillary electrophoresis (Haslam et al., 2006; Nakano et al., 2011; Yamaguchi and Kato, 2010). Recently, a lectin-based glycan profiling technology called lectin microarray has played a pivotal role in surveying and mapping the informational context of complex glycans of various biological samples, indicating the applicability of lectin-based glycan profiling (Hirabayashi et al., 2013; Narimatsu et al., 2010, 2018; Ribeiro and Mahal, 2013). However, there are limitations to the current glycan analytical methods. For instance, (1) glycans are unable to be analyzed at a single-cell level, (2) the glycan profile of each cell type in the mixed cell populations cannot be obtained without prior cell separation, and (3) the relationship between the glycome and transcriptome in single cells cannot be analyzed. Simultaneous analysis of the two modalities in single cells could lead to the understanding of cellular heterogeneity and glycan functions and the development of glycan markers of rare cells.

High-throughput single-cell sequencing has been transformative to understand the complex cell populations (Stuart and Satija, 2019). Recently, simultaneous profiling of multiple types of molecules within a single cell has been developed for building a much more comprehensive molecular view of the cell (Peterson et al., 2017; Stoeckius et al., 2017; Stuart and Satija, 2019). However, there has been no technology to jointly analyze the glycome and transcriptome in single cells since glycans cannot be amplified by polymerase chain reaction (PCR), unlike DNA and RNA. Here, we first established highly multiplexed lectin-based glycan profiling of single cells by sequencing (scGlycan-seq). We then combined the scGlycan-seq with single-cell transcriptome profiling (scRNA-seq) for joint analysis of glycan and RNA in single cells (scGR-seq). Using scGR-seq, we analyzed the relationship between the two distinct layers in human induced pluripotent stem cells (hiPSCs) before and after differentiation into neural progenitor cells (NPCs) in single cells and revealed the cellular heterogeneity across mRNA and glycans even within cells of the same cell types.

## Results

### Principle of Glycan-seq

**Figure 1:**
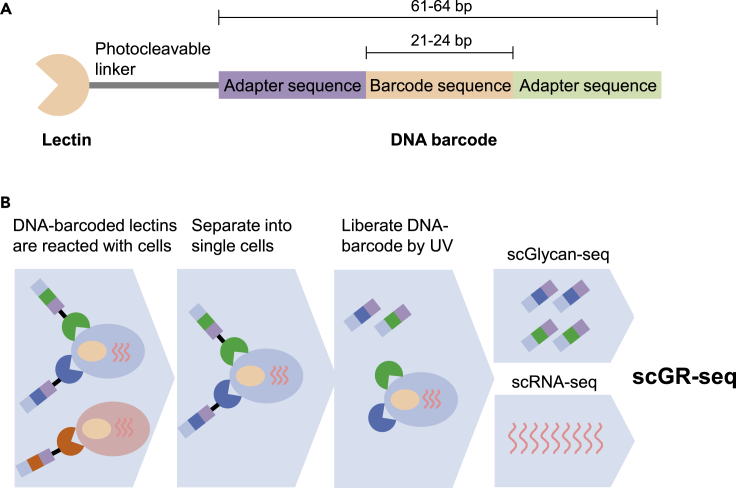
Principle of scGlycan-seq and scGR-seq (A) Illustration of the DNA-barcoded lectin. (B) Schematic representation of scGlycan-seq, scRNA-seq, and scGR-seq. Cells were incubated with DNA-barcoded lectins and separated into single cells. After DNA barcode was released by UV exposure, the released DNA barcode in the supernatants was measured by next-generation sequencing. RNA transcripts were purified from the cell pellet and analyzed by scRNA-seq. See also Figures S1–S3.

We hypothesized that a lectin conjugated with a DNA oligonucleotide (DNA-barcoded lectin) could be measured by sequencing as a digital readout of glycan abundance. To address this possibility, we conjugated lectins to DNA oligonucleotides designed to contain a barcode sequence for the identification of lectin and allowed specific identification by PCR (Figure 1A). Lectins were conjugated via their amino groups with photocleavable dibenzocyclooctyne-N-hydroxysuccinimidyl ester, which allowed efficient conjugation with 5′-azide-modified oligonucleotides (Figure S1). The lectin-to-oligonucleotide ratio was confirmed by measuring the concentration of DNA and lectin (Table S1). The oligonucleotides were released from the lectin by 15 min of ultraviolet (UV) exposure. Since the liberated amount of DNA increased as the UV exposure time by qRT-PCR (Figure S2), we determined the UV exposure time to balance the liberated amount of DNA and cell damage by UV exposure (Dion et al., 2019). DNA-barcoded lectins were then purified by affinity chromatography to remove excess DNA oligonucleotides. We prepared a panel of 39 DNA-barcoded lectins that can cover various glycans such as sialylated, galactosylated, GlcNAcylated, and mannosylated glycans displayed on glycoconjugates such as glycoproteins, glycolipids, and glycosaminoglycans in any organisms (Hsu et al., 2006; Krishnamoorthy et al., 2009; Tateno et al., 2011; Yasuda et al., 2011), and DNA-barcoded mouse and goat IgG was used as negative controls (Table S1). Total 41 DNA-barcoded proteins were incubated with 1 x 10^5^ cells, and unbound lectins were removed by washing (Figure 1B). Then, single-cell or 1 x 10^4^ cells (bulk) were separated into a PCR tube and exposed to UV. We picked up morphologically high-quality cells by visual inspection. After centrifugation, supernatants containing released DNA barcodes were recovered, amplified by PCR, and analyzed by next-generation sequencing (NGS) to count the DNA barcodes. We designated this method as glycan profiling of cells by sequencing (Glycan-seq).

To evaluate the PCR amplification bias of DNA barcodes, we prepared a mix of equal amount of 41 DNA barcodes used for the conjugation with 41 probes and performed 20 cycles of PCR followed by sequencing. The barcode counts for each DNA barcode were divided by those of all DNA barcodes and expressed as percentage (%). The average percentage was 2.43%, and the variation of the detected DNA barcodes ranged between 0.26 and 1.58-fold of the average percentage (Figure S3), suggesting that the PCR bias is less than 2 PCR cycles. Therefore, we considered that the PCR bias is within the allowable range since the purpose of the developed method is to compare the lectin binding signals between samples prepared with the same lot of DNA-barcoded lectin library.

### Glycan-seq of bulk cell populations

We assessed the ability of Glycan-seq to discriminate distinct cell populations based on cell surface glycan expression in bulk samples. Obtained data were compared with flow cytometry using fluorescence-labeled lectins as the gold standard.

**Figure 2:**
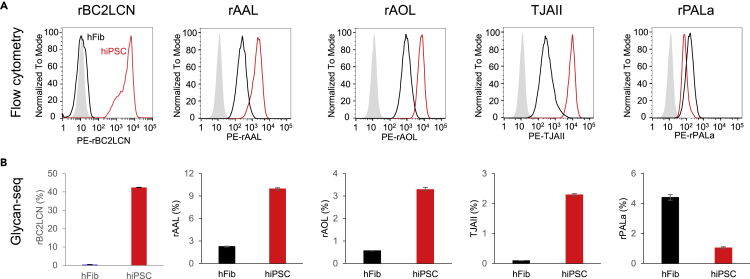
Comparison of Glycan-seq and flow cytometry (A) Binding of R-phycoerythrin (PE)-labeled lectins to hiPSCs (*red line*) and hFibs (*black line*) was analyzed by flow cytometry. *Gray*: Binding of PE-labeled BSA to hiPSCs (negative control). (B) Binding of DNA-barcoded lectins to hiPSCs and hFibs was analyzed by Glycan-seq. The number of DNA barcode derived from lectin was divided by that of the DNA barcode of all lectins, multiplied by 100, and expressed as percentage (%). Data are shown as average ± SD of triplicates of the same sample.

We first applied Glycan-seq to hiPSCs and human dermal fibroblasts (hFibs) with triplicates. A higher percentage of DNA-barcoded rBC2LCN, known to specifically bind to hiPSCs but not to hFibs(Onuma et al., 2013; Tateno et al., 2011, 2013), was detected in hiPSCs (42.4%) than in hFibs (0.4%), which was consistent with flow cytometry results (Figure 2, Tables S2 and S3). Mouse and sheep IgGs were used as negative controls, providing negligible DNA barcode levels (<0.01%) (Tables S2 and S3). Glycan-seq data of other lectins such as fucose binders (rAAL, rAOL, TJAII) and a mannose binder (rPALa) also agreed well with flow cytometry data (Figure 2).

**Figure 3:**
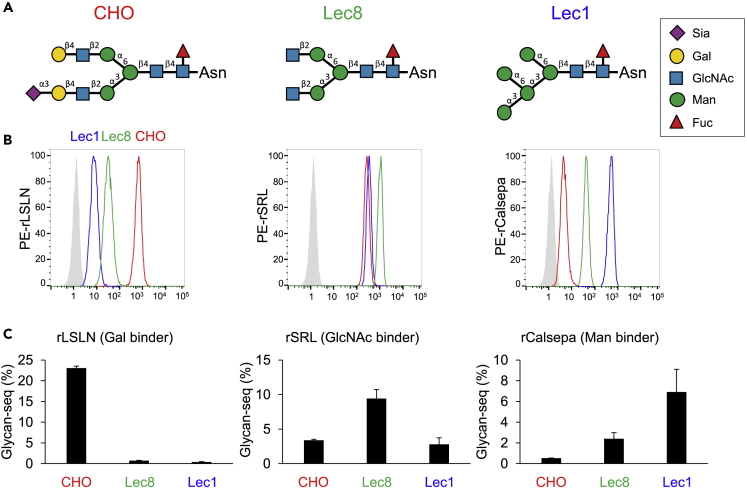
Glycan-seq analysis of CHO, Lec8, and Lec1 (A) Typical N-glycan structures expressed in CHO, Lec8, and Lec1. (B) Binding of Gal-binder (rLSLN), GlcNAc-binder (rSRL), and Man-binder (rCalsepa) to CHO, Lec8, and Lec1 analyzed by flow cytometry. (C) Binding of Gal-binder (rLSLN), GlcNAc-binder (rSRL), and Man-binder (rCalsepa) to CHO, Lec8, and Lec1 analyzed by GR-seq. Data are shown as average ± SD of triplicates of the same sample.

We next applied Glycan-seq to Chinese hamster ovary (CHO) cells and glycosylation-defective mutants (Lec1 and Lec8) (Figure 3, Tables S2 and S3) (Esko and Stanley, 2015). Wild-type (WT) cells typically express complex-type N-glycans (North et al., 2010), while Lec8 and Lec1 express agalactosylated and mannosylated N-glycans, respectively. In both flow cytometry and Glycan-seq, rLSLN (galactose binder) showed higher binding to WT than Lec8 and Lec1. Similarly, rSRL (GlcNAc binder) and rCalsepa (mannose binder) showed strong binding to Lec8 and Lec1, respectively. These results demonstrated that Glycan-seq could distinguish different bulk cell populations depending on cell surface glycan expression.

**Figure 4:**
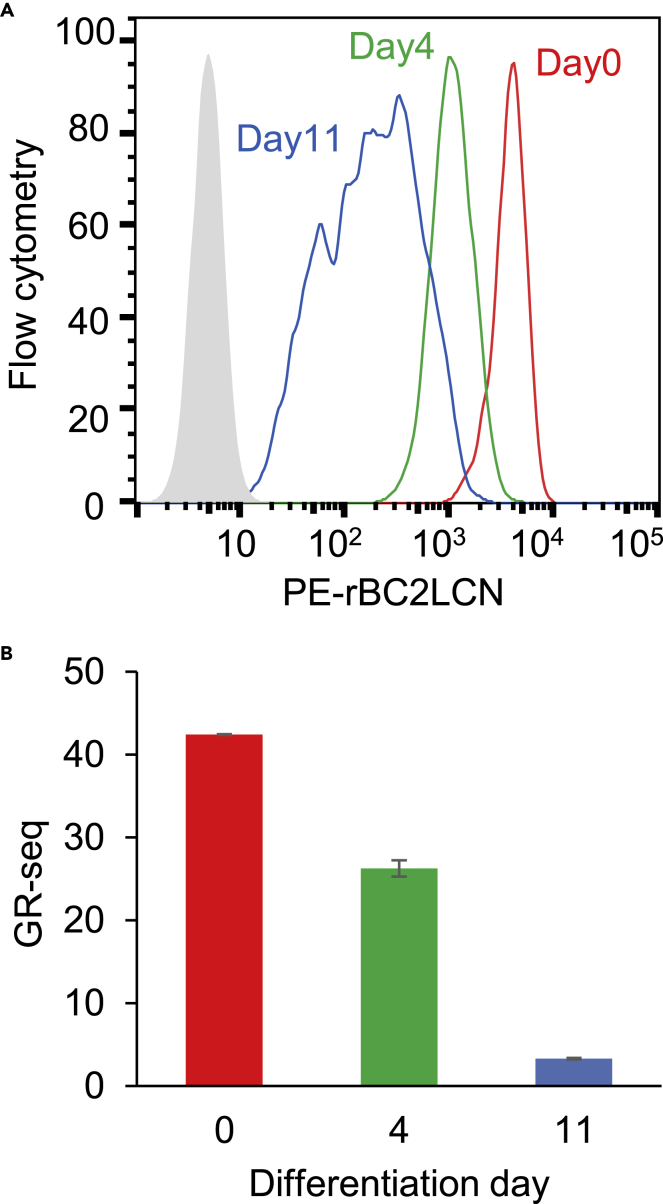
Glycan-seq analysis of hiPSC and NPCs (A) Binding of PE-labeled rBC2LCN to hiPSCs after differentiation to NPCs for 0 (*red line*), 4 (*green line*), and 11 days (*blue line*) was analyzed by flow cytometry. (B) Binding of DNA-barcoded rBC2LCN to hiPSCs after differentiation to NPCs for 0 (*red bar*), 4 (*green bar*), and 11 days (*blue bar*) was analyzed by Glycan-seq. Data are shown as average ± SD of triplicates of the same sample. See also Figures S4–S6.

We further addressed whether relative quantitative differences in expression levels observed by flow cytometry could be measured by Glycan-seq (Figure 4, Tables S2 and S3). We applied Glycan-seq to hiPSCs before (day 0) and after (day 4 and 11) differentiation to NPCs, which were fluorescently stained with NPC marker (NESTIN and PAX6) antibodies in fluorescence staining and flow cytometry (Figures S4 and S5). Using qRT-PCR, NPC marker genes (*SOX1*, *NESTIN*, *PAX6*, *FOXG1*), but not an hiPSC marker gene (*OCT4*) (Obernier and Alvarez-Buylla, 2019; Takahashi et al., 2007), were detected in NPCs (Figure S6), indicating that hiPSCs could be successfully differentiated into NPCs. In flow cytometry, rBC2LCN binding gradually decreased during differentiation into NPCs (Figure 4A). Similar trends were observed for rBC2LCN signal in Glycan-seq (Figure 4B, Tables S2 and S3). Collectively, these results indicate that bulk Glycan-seq can capture distinct and quantitative differences in glycan profiles in various cell populations as observed by flow cytometry.

### Single-cell Glycan-seq

We then tested Glycan-seq to see its applicability in single cells, which we termed glycan profiling of single cells by sequencing (scGlycan-seq) (Figure 1B). We first applied scGlycan-seq to hiPSCs and hFibs. To assess the effect of the total barcode counts in each cell on the obtained glycan profiles, we performed principal component analysis (PCA) on the scGlycan-seq data. Single cells of hFibs with low total barcode counts showed low values in PC1 (Figure S7A), suggesting total barcode counts were a confounding factor. Since the cells with low and high total barcode counts were separated in the histogram (Figure S7B), we sought to remove cells with low total barcode counts from the downstream analyses and determined the cut-off value of 19,465 total barcode counts by Otsu's method. After the removal of hFibs with low total barcode counts, PCA showed no association of the total barcode counts with PC1 and PC2 (Figure S7C), indicating that cells were no longer biased by total barcode counts. The same quality control was adapted for hiPSCs, and hiPSCs with higher than 6,126 total counts were used for the following analysis.

**Figure 5:**
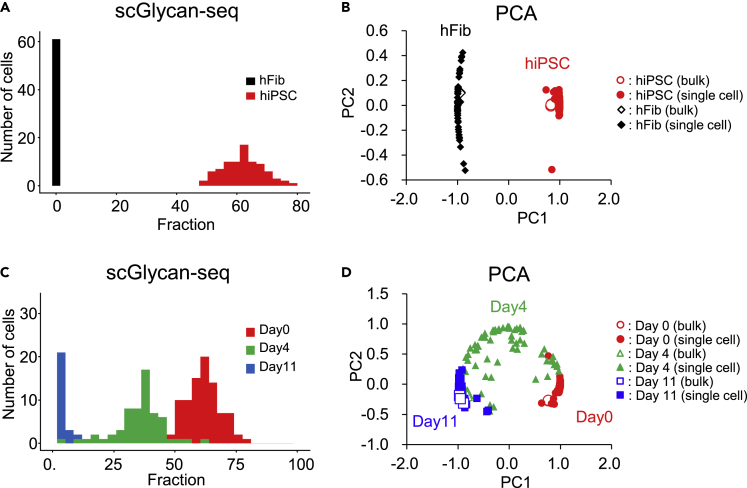
scGlycan-seq analysis of hiPSCs, hFibs, and NPCs (A) Binding of DNA-barcoded rBC2LCN to hiPSCs and hFibs analyzed by scGlycan-seq. The histogram of rBC2LCN signal levels measured for hiPSCs (n = 83, *red*) and hFibs (n = 61, *black*) is shown. There was statistical significance in the distribution of rBC2LCN between the two cell types (p < 0.001, Brunner-Munzel test with Bonferroni correction). (B) PCA of bulk (100 cells, n = 3 for each cell type) and single cells (n = 96 for each cell type) of hiPSCs (*filled circle*: single cell, *open circle*: bulk) and hFibs (*filled triangle*: single cell, *open triangle*: bulk). PC1: principal component 1. PC2: principal component 2. (C) Binding of DNA-barcoded rBC2LCN to hiPSCs after differentiation to NPCs analyzed by scGlycan-seq. The histogram of rBC2LCN signal levels measured for hiPSCs after differentiation to NPCs for 0 (n = 84, *red*), 4 (n = 61, *blue*), and 11 days (n = 57, *green*) is shown. There was statistical significance in the distribution of rBC2LCN between the two cell types (day 0 vs day 4; p < 0.001, day 4 vs day 11; p < 0.001, day 0 vs day 11; p < 0.001, Brunner-Munzel test with Bonferroni correction). (D) PCA of bulk (100 cells, n = 3 for each cell type) and single cells (n = 96 for each cell type) of hiPSCs after differentiation to NPCs for 0 (*open circle*: bulk, *filled circle*: single cell), 4 (*open triangle*: bulk, *filled triangle*: single cell), and 11 days (*open square*: bulk, *filled square*: single cell). DNA barcode derived from each lectin was divided by DNA barcode of all lectins and multiplied by 100. Glycan-seq data are available in Tables S2–S5. PC1: principal component 1. PC2: principal component 2. See also Figures S7 and S8.

The signal level of rBC2LCN in hiPSCs and hFibs obtained by scGlycan-seq was shown in Figure 5A. scGlycan-seq recapitulated heterogeneity of rBC2LCN observed by flow cytometry and showed statistically significant differences between hiPSCs and hFibs (p < 0.001, Brunner-Munzel test). We then performed PCA on scGlycan-seq data together with bulk Glycan-seq data of hiPSCs and hFibs (Figure 5B). The PC1 clearly separated the two cell types, and, for each of hiPSCs and hiFibs, the PC2 showed higher variability of single cells compared to bulk samples, revealing cell-to-cell heterogeneity in glycan profiles (Figure 5B, Tables S4 and S5).

We further applied scGlycan-seq to the hiPSCs after differentiation into NPCs (days 0, 4, and 11). The signal level of each lectin in hiPSCs and 11-day NPCs was shown in Figure S8. Relative quantitative differences in the rBC2LCN signal for hiPSCs before (day 0) and after differentiation to NPCs (day 4 and 11) observed by flow cytometry could also be captured by scGlycan-seq (Figure 5C). Statistically significant differences between hiPSCs (day 0) and hiPSC-derived NPCs (day 4 and 11) (p < 0.001, Brunner-Munzel test) were observed. PCA clearly separated single cells of day 0, 4, and 11, and cells were ordered as differentiation progression (Figure 5D). Single-cell heterogeneity of hiPSCs increased after 4-day differentiation but converged after 11-day differentiation, possibly because 4-day NPCs might contain various degrees of differentiated NPCs (Figure 5D, Tables S4 and S5). These results demonstrated that scGlycan-seq enabled glycan profiling in single cells and revealed cellular heterogeneity in glycan profiles.

### scGR-seq of hiPSCs and NPCs

We combined scGlycan-seq with scRNA-seq to enable the simultaneous measurement of the glycome and transcriptome in single cells, termed scGR-seq (Figure 1B). Specifically, we employed RamDA-seq, a full-length single-cell total RNA-sequencing method^7^. We performed scGR-seq on the hiPSCs (n = 53) and hiPSC-derived NPCs (11-day differentiation) (n = 43) (Tables S6–S9). After quality control of scRNA-seq data of hiPSCs and NPCs (see STAR Methods and Figures S9–S12), we searched for differentially expressed genes between hiPSCs and NPCs. We found that 1,131 and 688 genes were significantly upregulated in hiPSCs and NPCs, respectively (Figure S13, Table S10). Consistent with neural differentiation of hiPSCs, GO enrichment analysis demonstrated that gene sets annotated with neuron-associated terms were significantly enriched in NPCs (Table S11). Furthermore, transcriptome data of hiPSCs and hiPSC-derived NPCs showed cell-type-specific expression of 41 selected cell-type marker genes (Figure S14) (Obernier and Alvarez-Buylla, 2019; Takahashi et al., 2007). For example, NPCs showed higher expression of neural progenitor marker genes such as *NES* (*NESTIN*), *PAX6*, and *SOX1* (Obernier and Alvarez-Buylla, 2019) and lower expression of hiPSC marker genes such as *NANOG* and *POU5F1*(Takahashi et al., 2007), which agree well with fluorescence staining (Figure S4). These data suggest that the scRNA-seq data of GR-seq reflect transcriptome information with biological relevance.

**Figure 6:**
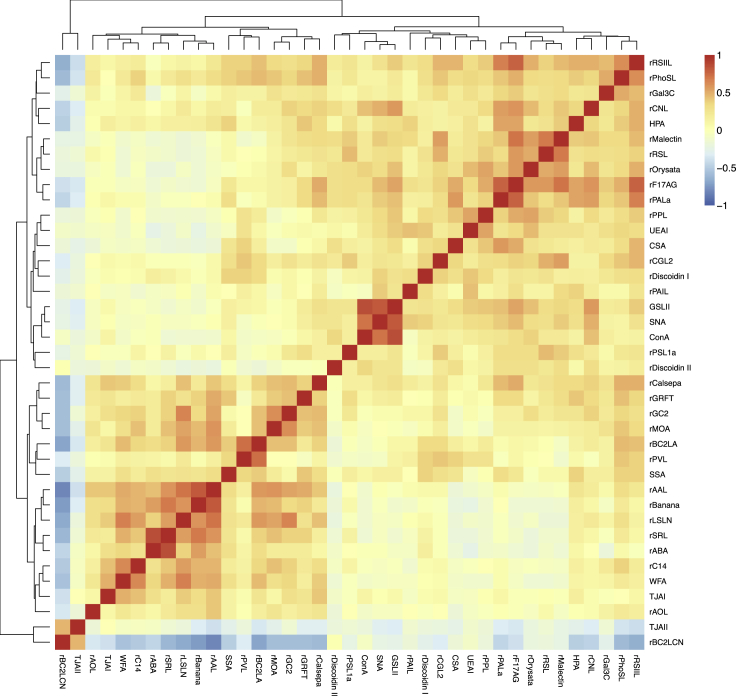
Correlation of lectins across cells A heatmap shows the Pearson correlation coefficient of each pair of lectins. scGlycan-seq data of hiPSCs (n = 53) and NPCs (n = 43) were used. Rows and columns represent lectins.

We analyzed the correlation of lectins across cells and found lectins that fluctuated with each other based on their correlation (Figure 6). Lectins were separated into two large clusters: one cluster containing rBC2LCN and TJAII and another cluster containing other 37 lectins. rBC2LCN and TJAII commonly recognize α1-2fucosylated glycans, which are upregulated in hiPSCs (Figures 6 and S8) (Hasehira et al., 2012; Sulak et al., 2010; Tateno et al., 2011; Yamashita et al., 1992). In contrast, other lectins showed higher binding to NPCs or comparable signals between the two cell types (Figures 6 and S8).

**Figure 7:**
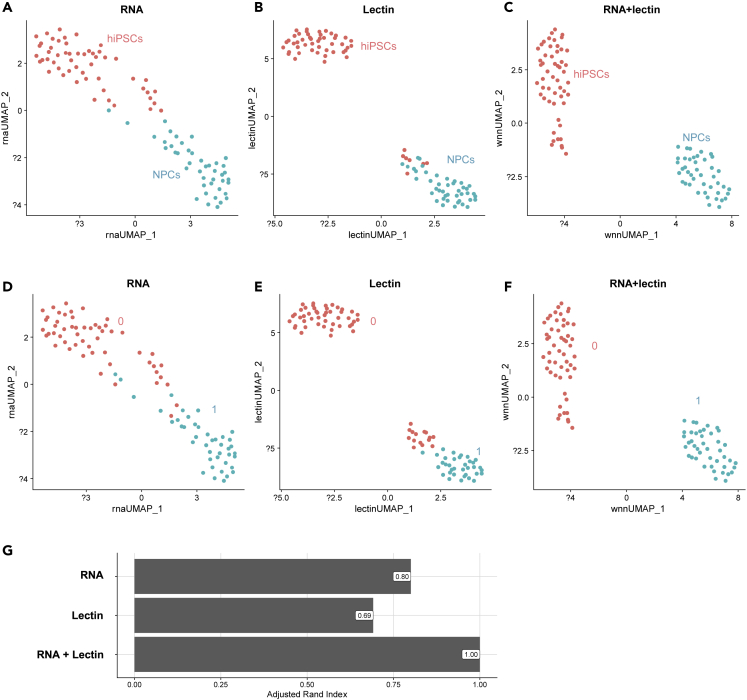
Dimensional reduction and clustering using the Seurat workflow (A–C) UMAP visualization based on (A) only the scRNA-seq data and (B) only the scGlycan-seq, and (C) both scRNA-seq and scGlycan-seq (scGR-seq) data of hiPSCs (n = 53, *red*) and NPCs (n = 43, *green*). UMAP computation was performed based on the weighted nearest neighbor graph that integrates the RNA and lectin modalities. (D–F) Same UMAP visualization as (A-C) but single cells are colored by the clustering results based on (D) only scRNA-seq data, (E) only scGlycan-seq data, and (F) both scRNA-seq and scGlyca-seq (scGR-seq) data using a shared nearest neighbor (SNN) modularity optimization-based clustering algorithm. (G) Bar plots showing adjusted Rand index as a measure of the similarity of the clustering in (D), (E), and (F) to the cell type annotation (A–C). See also Figures S9–S13.

We then addressed how the information from two modalities can be used. When we performed Uniform Manifold Approximation and Projection (UMAP), a non-linear dimensional clustering, based on only the mRNA or glycan data using the Seurat workflow, the two cell types (hiPSCs and NPCs) were partially separated (Figures 7A and 7B)*.* In contrast, when we performed UMAP based on both the mRNA and glycan data using the Seurat-weighted nearest neighbor workflow, the two cell types were clearly separated (Figure 7C). We also found that the unsupervised clustering using both mRNA and glycan data completely agreed with the cell type annotation (Figure 7F), whereas the clustering based on either mRNA or glycan data showed poorer concordance (Figures 7D and 7E). The difference in the clustering results was quantitatively verified by the adjusted Rand index (Figure 7G). No bias was observed by the number of genes or lectins on UMAP (Figure S15). This tendency was also confirmed when we performed PCA based on the mRNA or lectin data and partial least squares (PLS) regression using both mRNA and glycan information, where the latter separated the two cell types clearer than the former two (Figure S16). These results demonstrated how the combination of mRNA and glycan modalities help characterize cell identities.

### Relationship between mRNA and glycan in single cells

**Figure 8:**
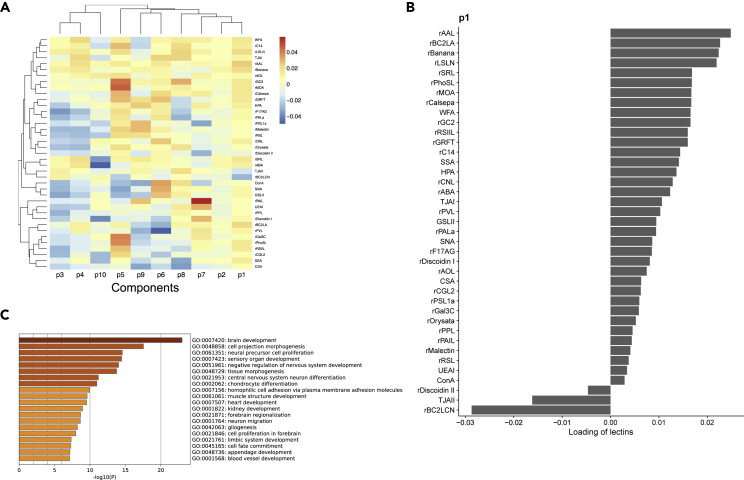
PLS regression (A) A heatmap of the association between each lectin and each component inferred by PLS regression. Rows represent lectins and columns represent components. (B) Weights of lectins for the component p1 from the PLS regression results. (C) Enriched gene sets for the genes associated with the component p1 of the PLS regression results. Gene enrichment analysis was performed with Metascape. The results of gene enrichment analysis are available in Table S12. See also Figure S15.

Simultaneous transcriptome and glycome measurements could associate genes with glycans at the single-cell level. The PLS regression analysis described above found a group of mRNAs and lectins that were associated with each other differently per component (see STAR Methods, Figure 8A). For the component p1, where weights were high for rAAL, rBC2LA, rLSLN, and rBanana, the mRNAs (high p1 weight) related to brain development, cell projection morphogenesis, neural precursor cell proliferation, sensory organ development, and negative regulation of nervous system development were the most enriched gene sets (Figures 8B and 8C, Table S12), suggesting that the glycan ligands of these lectins might be closely associated with neural differentiation. Other components (p2-p10) also showed the relationship between lectins and the set of genes (Figures S17–S21). This analysis allowed us to infer each glycan's potential functions and roles as a marker through the set of genes associated with the glycan. Furthermore, by summing the loadings of each component, we obtained the overall relationship between lectins and glycosylation-related genes (see STAR Methods and Figure S22). For example, *ST6Gal1*, which catalyzes the synthesis of α2,6Sia, showed the highest correlation with α2-6Sia-binding lectin rPSL1a (Kadirvelraj et al., 2011; Tateno et al., 2004). These exemplify how scGR-seq is useful for finding potential relevance between transcriptome and glycome layers, although further detailed analysis should be performed in future studies.

### scGR-seq reveals pluripotency and differentiation glycan markers

**Figure 9:**
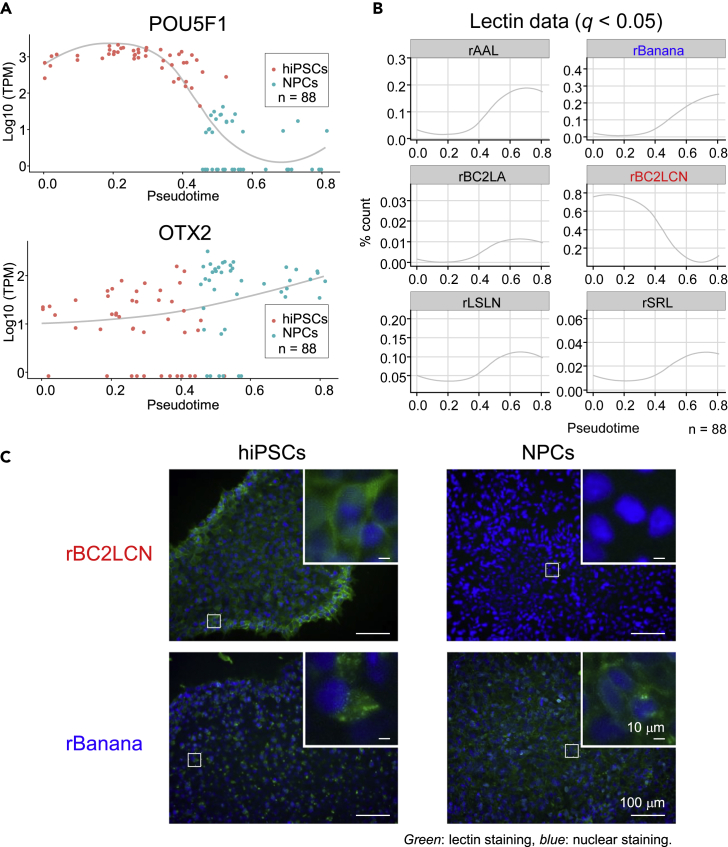
Psuedotime analysis of mRNA and lectin signals (A) Expression profile of the hiPSC marker (*POU5F1*) and NPC marker (*OTX2*) genes. The *x* axis represents the pseudotime inferred by slingshot using mRNA data. The *red* and *blue* points indicate hiPSCs and NPCs, respectively. The curve represents smoothed conditional means calculated by the LOESS (locally estimated scatterplot smoothing) method. (B) Dynamically regulated lectins along the pseudotime. The pseudotime is the same as in (A). A generalized additive model was fitted to the lectin signals to search for dynamically regulated lectins (*q* < 0.05 after the multiple testing correction using the Benjamini and Hochberg method). The *red* and *blue* points indicate hiPSCs and NPCs, respectively. The curve represents smoothed conditional means calculated by the LOESS method. (C) Fluorescence staining of hiPSCs and hiPSC-derived NPCs by rBC2LCN and rBanana. hiPSCs and hiPSC-derived NPCs (17-day differentiation) were fixed with 4% paraformaldehyde at room temperature for 20 min. After blocking with PBS containing 1% BSA at room temperature for 30 min, cells were incubated with 1 μg/mL of fluorescently labeled rBC2LCN and rBanana for 1 hr at room temperature and observed by fluorescence microscopy. The nucleus was stained with Hoechst 33,342 (x 1,000) at room temperature for 5 min. Insets show high magnification of selected fields. Nucleus: *blue*. lectin: *green*. Scale bar: 100 μm or 10 μm (insets). See also Figures S16 and S17.

A pseudotime analysis on the mRNA data of hiPSCs and NPCs reconstructed within-cell-type variability reflecting cell differentiation progression (see STAR Methods): expression of hiPSC (*POU5F1*) and NPC (*OTX2*) marker genes showed a progressive decrease and increase along with the pseudotime (generalized additive model, *q* < 0.05) (Figure 9A), indicating biological relevance of pseudotime reconstruction. Based on the mRNA pseudotime, we next searched for lectins showing changes along with the pseudotime (Figure 9B; generalized additive model, *q* < 0.05). rBC2LCN, the known pluripotency marker probe, was decreased along pseudotime, while other lectins such as rBanana (mannose binder), which is not known as any cell surface marker, were increased. To confirm the differential binding of rBC2LCN and rBanana, we conducted fluorescence microscopy examination. Consistently, rBC2LCN staining was diminished after differentiation into NPCs (Figure 9C). While rBanana showed intracellular organelle staining in hiPSCs, the cell surface was stained in hiPSC-derived NPCs, suggesting a drastic change in the localization of the glycan ligands of rBanana during differentiation (Figure 9C). Because rBanana showed high weight in component p1 (Figure 8C) and the component p1 was associated with neuron-related gene sets (Figure 8C), the glycan ligands of rBanana may be related to neural differentiation.

We also examined genes correlated with hiPSC-specific rBC2LCN at the single-cell level (Figure S23 and Table S13). The highest positive correlation coefficient (0.77) was observed with the hiPSC marker gene (*POU5F1*), whereas the highest negative correlation coefficient (−0.65) was observed with the NPC marker gene (*VIM*), supporting the previous finding that rBC2LCN was a maker probe for hiPSCs (Tateno et al., 2011).

Furthermore, we searched for lectins correlated with hiPSC marker genes (*MYC*, *LEFTY1*, and *LEFTY2*) within hiPSCs (Figure S24), all of which are known to show gene expression variability in human pluripotent stem cells. Expectedly, rBC2LCN exhibited the highest correlation with *MYC*. *LEFTY1* and *LEFTY2* exhibited the highest correlation with rGal3C (galactose binder) and rBC2LA (mannose binder), respectively, suggesting a possible link between glycan ligands of these lectins and the functions of *LEFTY1* and *LEFTY2* in the regulation of self-renewal and differentiation (Kim et al., 2014; Tabibzadeh and Hemmati-Brivanlou, 2006; Takahashi et al., 2007). These results demonstrate that scGR-seq revealed the coordinated heterogeneity in pluripotency/differentiation markers across mRNA and glycans even within cells of the same cell types.

## Discussion

In this study, we first established glycan profiling by sequencing (Glycan-seq) using 41 DNA-barcoded proteins. Distinct cell populations could be discriminated based on cell surface glycan expression. Relative quantitative differences in expression levels of glycans observed by flow cytometry could be measured by Glycan-seq. No visible non-specific interaction between lectins was observed under the condition used in this study. Furthermore, Glycan-seq was proved to be applicable to single cells. Therefore, Glycan-seq is feasible for profiling cell surface glycans in both bulk and single cells. The amount of DNA barcodes conjugated with lectins affects the lectin binding signals. However, the aim of the developed method is to compare the lectin binding signals between samples by using the same lot of DNA-barcoded lectin library, which is similar to flow cytometry analysis, other sequencing-based analytical methods such as CITE-seq (Stoeckius et al., 2017), and lectin microarray(Hirabayashi et al., 2013).

We then combined scGlycan-seq with RamDA-seq (full-length total RNA sequencing in single cells), which can analyze not only mRNA but also non-poly(A) RNAs such as nascent RNAs, histone mRNAs, long noncoding RNAs (lncRNAs), circular RNAs (circRNAs), and enhancer RNAs (eRNAs) (Hayashi et al., 2018). Although we focused on the relationship between glycan and mRNA in this study, it would be interesting to analyze the relevance of glycan with non-poly(A) RNA at the single-cell resolution in future studies.

Using scGR-seq, we analyzed the two modalities in hiPSCs before and after differentiation into neural progenitor cells. The combination of RNA and glycan separated the two cell types clearer than either one of them. Furthermore, integrative analysis of glycan and RNA modalities in single cells found known and unknown lectins that were specific to hiPSCs and coordinated with neural differentiation. rBC2LCN showed the highest correlation with the well-known pluripotency marker gene, *POU5F1* among all genes expressed in hiPSCs and NPCs, further supporting the previous finding that rBC2LCN signal is associated with pluripotency (Hasehira et al., 2012; Onuma et al., 2013; Tateno et al., 2011, 2013, 2015). rBanana with specificity to mannosylated glycans was extracted as a lectin to increase along with neural differentiation by PLS regression (Figure 8) and pseudotime analysis (Figure 9B). Indeed, rBanana showed cell surface staining to NPCs, while its staining was observed in cytoplasm in hiPSCs (Figure 9C). We performed two independent experiments for the analysis of hiPSCs and NPCs in single cells and basically obtained similar results. This suggests that the plate effects (plate-to-plate variability) are visually low. These results demonstrate that mannosylated glycans might increase at the surface of NPCs, which could be used as a cell surface marker for identification and separation of NPCs and might lead to the understanding the functions of NPCs.

In terms of glycan detection probes, cost-effective and commercially available lectins, either from natural sources or recombinantly expressed, were used for Glycan-seq (Hirabayashi et al., 2013; Ribeiro and Mahal, 2013). Any glycan-binding probes, such as anti-glycan antibody and glycan-binding peptide, can be incorporated in Glycan-seq (McKitrick et al., 2020).

scGR-seq can be widely adapted to cells, organoids, and tissues and can be applied to various scientific fields such as stem cell biology, immunology, cancer biology, and neuroscience. One of the biggest advantages of scGR-seq is that the method could be applied for the development of glycan markers of rare cells such as circulating tumor cells, fetal nucleated red blood cells, and cancer stem cells. Identification of glycan markers of rare cells could be performed as follows: (1) cell populations such as tissues, organoids, and hiPSC-derived cells/organs are analyzed by scGR-seq. (2) Lectins with high intensity to rare cells identified by transcriptome data are selected. Such lectins might be directly used for the identification and concentration of rare cells. (3) Glycoprotein ligands of lectins expressed in rare cells are identified by lectin pull down followed by LS-MS/MS. (4) Monoclonal antibodies recognizing both a glycan epitope and a peptide sequence could be generated as specific probes for rare cells (Watanabe et al., 2020). Such antibodies would be useful for the development of diagnosis (Egashira et al., 2019) and antibody drugs (Kato and Kaneko, 2014). scGR-seq can also be applied to cells derived from other organisms such as fungi and bacteria using the available platform, as lectins can bind glycans expressed by the cells of any organism (Hsu et al., 2006; Yasuda et al., 2011).

Recently, SUrface-protein Glycan And RNA-seq (SUGAR-seq) based on the 10x Genomics platform was reported that enables detection of a lectin-binding signal together with the analysis of extracellular epitopes and the transcriptome at the single-cell level (Kearney et al., 2021). However, SUGAR-seq detects only one lectin binding signal to single cells. In this regard, scGR-seq can acquire 39 lectin-binding signals to single cells, which can capture a whole picture of cell surface glycans (Table S14). It should be noted that the number of lectins is expandable in scGR-seq. We believe that scGR-seq has the potential to advance the understanding of cellular heterogeneity and the biological roles of glycans across diverse multicellular systems across species.

### Limitations of study

There are limitations in scGlycan-seq and scGR-seq. Similar to flow cytometry and lectin microarray, absolute amounts of glycans and accurate glycan structures cannot be determined directly from the signal intensities as described above. Another limitation of the current system is the throughput. Since scGR-seq is a plate-based platform, processing of cell numbers is limited to hundreds of cells, while it can perform full-length total RNA sequencing (Table S14) (Hayashi et al., 2018). In contrast, droplet-based methods such as 10x Genomics (CITE-seq) can sequence thousands of cells at once but target only the 3′ends of poly(A) transcripts (Baran-Gale et al., 2018). Due to this difference, scGR-seq will complement for studying single cells in complex biological systems. To solve this limitation, scGR-seq should be adapted to droplet (Bageritz and Raddi, 2019), nano-well(Gierahn et al., 2017), and indexing-based high-throughput single-cell technologies (Rosenberg et al., 2018) in future studies.

## STAR★Methods

### Key resources table

**Table:** 

REAGENT or RESOURCE	SOURCE	IDENTIFIER
**Antibodies**		

PE Mouse anti-Human Pax-6	BD Biosciences	clone No. O18-1330; RRID:AB_10715442
PE Mouse anti-Nestin	BD Biosciences	clone 25/NESTIN (RUO); RRID:AB_10562398

**Chemicals, peptides, and recombinant proteins**		

mTeSR Plus	VERITAS	ST-100-0276
Matrigel	CORNING	REF 354230
MesenPRO RS medium	Thermo Fisher Scientific KK	12746012
Dibenzocyclooctyne-N-hydroxysuccinimidyl ester	Funakoshi Co., Ltd	A133-25

**Critical commercial assays**		

Bradford protein assay	Bio-Rad Laboratories	5000001JA
Quant-iT OliGreen ssDNA Reagent	Thermo Fisher Scientific KK	O7582
PowerUp SYBR Green Master Mix	Thermo Fisher Scientific KK	A25741
STEMdiff SMADi Neural Induction Kit	VERITAS	CATALOG #08581
NEBNext Ultra II Q5 Master Mix	New England BioLabs Japan Inc	M0544S
Agencourt AMPure XP kit	Beckman Coulter, Inc.	BC-A63880
Miseq Reagent Kit v2 50 Cycles	Illumina KK	MS-102-2001
GenNext RamDA-seq Single Cell Kit	TOYOBO	RMD-101
R-phycoerythrin Labeling Kit	Dojindo Laboratories Co. Ltd.	LK23
RNeasy Mini Kit	QIAGEN	Cat. no.74104

**Deposited data**		

All raw data of scRNA-seq	This paper	GSE151642

**Experimental models: Cell lines**		

Human: iPS cell line 201B7	RIKEN Bio Resource Center	HPS0063
Human: Primary Dermal Fibroblast	ATCC	PCS-201-012
Chinese hamster: CHO cell line CHO-K1	ATCC	CCL-61
Chinese hamster: CHO cell line Lec1	ATCC	CRL-1735
Chinese hamster: CHO cell line Lec8	ATCC	CRL-1737

**Software and algorithms**		

Barcode DNA counting system	This paper	N/A
R version 3.6.1	The R Foundation	https://www.r-project.org/

**Other**		

TOPick I Live Cell Pick system	Yodaka Giken	N/A

### Resource availability

#### Lead contact

Further information and requests for resources and reagents should be directed to and will be fulfilled by the lead contact, Hiroaki Tateno (h-tateno@aist.go.jp).

#### Materials availability

Materials generated in this study can be requested from the lead contact.

### Experimental model and subject details

201B7 hiPSCs were obtained from RIKEN Bio Resource Center and cultured in mTeSR Plus medium (VERITAS, Tokyo, Japan) on a Matrigel (Corning International, Tokyo, Japan) coated plates. 201B7 hiPSCs were differentiated to neural progenitor cells using STEMdiff SMADi Neural Induction Kit (VERITAS).

### Methods details

#### Cells

Human primary dermal fibroblasts were purchased from ATCC (Manassas, Virginia) and cultured in MesenPRO RS medium (Thermo Fisher Scientific KK).

CHO cells and its glycosylation-defective mutants (Lec1 and Lec8) were purchased from ATCC (Manassas, Virginia) and cultured in DMEM medium supplemented with 10% heat-inactivated fetal bovine serum, 2 mM glutamine, and 25 mM HEPES (pH 7.4).

#### Conjugation of lectins to DNA oligonucleotides

100 μg of lectin in 100 μl of PBS was incubated with ten-times the molar amount of dibenzocyclooctyne-N-hydroxysuccinimidyl ester (DBCO-NHS) (Funakoshi Co., Ltd., Tokyo, Japan) at 20°C for 1 h under dark (Figure S1). 10 μl of 1 M Tris was then added and incubated at 20°C for 15 min under dark to inactivate the excess DBCO-NHS. The excess DBCO-NHS was also removed by using Sephadex G-25 desalting columns (GE Healthcare Japan Co., Tokyo, Japan). The resulting DBCO-labeled lectin was then incubated at 4°C with ten-times the molar amount of 5’-azide-modified DNA oligonucleotides (Integrated DNA Technologies, KK, Tokyo, Japan). To remove unbound oligonucleotides and obtain lectins with sugar-binding activity, the DNA-barcoded lectin was purified by affinity chromatography using appropriate sugar-immobilized Sepharose 4B-CL (GE) based on the glycan-binding specificity of each lectin. Finally, DNA-barcoded lectin was dialyzed by Tube-O-DIALYZER (Takara Bio Inc., Shiga, Japan) and concentrated using centrifugal filters having a 10 kDa molecular weight cut off (MWCO) (Merck KGaA, Darmstadt, Germany). The purified DNA-barcoded lectin was analyzed by SDS-PAGE. Protein and DNA concentration were measured using the Bradford protein assay (Bio-Rad Laboratories, Inc., CA, USA) and Quant-iT OliGreen ssDNA Reagent (Thermo Fisher Scientific KK, Tokyo, Japan).

#### List of lectins

See Table S1 for a list of lectins and barcodes used for bulk and single cell Glycan-seq and GR-seq.

#### Glycan-seq in bulk and single cells

Cells (1x10^5^) were suspended in phosphate-buffered saline (PBS) containing 1% bovine serum albumin (BSA) and incubated with 41 DNA-barcoded proteins at a final concentration of 0.5 μg ml^-1^ at 4°C for 1 h. After washing three times with 1 ml of PBS/BSA, cell number was counted by TC20 auto cell counter (Bio-Rad Laboratories, Inc.). Cells (1x10^4^ cells per tube or 1 cell per tube) were distributed into a PCR tube (FCR&BIO Co., LTD., Hyogo, Japan). One cell dispense was performed using TOPick I Live Cell Pick system (Yodaka Giken, Kanagawa, Japan). To liberate oligonucleotides from cells, the cells were irradiated at 365 nm, 15 W for 15 min using UVP Blak-Ray XX-15L UV Bench Lamp (Analytik Jena, Kanagawa, Japan).

The liberated oligonucleotides were then amplified using NEBNext Ultrall Q5 (New England BioLabs Japan Inc., Tokyo, Japan), and i5-index and i7-index primers containing cell barcode sequences (Table S15). PCR reactions were performed as follows: denaturing (45s at 98°C, 1 cycle), amplification (10s at 98°C, 50 s at 65°C, 20 cycles), extension (5 min at 65°C, 1 cycle). The PCR products were then purified by the Agencourt AMPure XP kit (Beckman Coulter, Inc., Tokyo, Japan), followed by the manufacturer’s protocol. The size and the quantity of the PCR products were analyzed by MultiNA (Shimadzu Co., Kyoto, Japan). The PCR products (4 nM from every single cell) were treated with the Miseq Reagent Kit v2 50 Cycles (Illumina KK, Tokyo, Japan) and sequenced by the MiSeq sequencer (26 bp, paired-end) (Illumina KK).

#### Data analysis of Glycan-seq

DNA barcodes derived from lectins were directly extracted from the reads in the FASTQ format. The number of DNA barcodes bound to each cell was counted using the developed software, Barcode DNA counting system (Mizuho Information & Research Institute, Inc., Tokyo, Japan). In order to match the sequence, the first three bases were removed. As maximum, two mismatches in flanking region and one mismatch in middle region were accommodated. Each DNA barcode count was divided by the total lectin barcode count and expressed as a percentage (%) for each lectin. In order to filter out the cells with low total barcode count, the cut-off value of total lectin barcode count was determined using Otsu's method(Otsu, 1979). The cut-off value was determined for each cell type, and hFibs = 19,465, hiPSCs = 6,126, Day4 = 14,723 and Day 11 = 8,798 were used for the analysis (Figure 5). No bias for principal component analysis (PCA) due to the total barcode count was observed for scGR-seq data of hiPSCs and NPCs. PCA was performed to simplify the multivariate data of glycan-seq by reducing the dimensionality. PCA was carried out by SPSS Statistics 19 (IBM Japan, Tokyo, Japan). Data were preprocessed using mean-centering prior to this analysis. Using the first two principal component (PCs), each sample data was visualized on two dimensional plot.

To test the difference of the rBC2LCN signal in scGlycan-seq between hiPSCs and hFibs, and hiPSCs and NPCs, we used the Brunner-Munzel test, an independent two-sample test that assumes neither normality nor homoscedasticity(Otsu, 1979), using the ‘brunner.munzel.test’ function in ‘lawstat’ R package.

#### scRNA-seq

Single-cell cDNA library was prepared using the GenNext RamDA-seq Single Cell Kit (Toyobo Co., LTD. Tokyo, Japan), followed by the manufacturer’s protocol(Hayashi et al., 2018). The obtained library was then quantified by real-time PCR using the PowerUp SYBR Green Master Mix (Thermo Fisher Scientific KK) and CFX Connect System (Bio-Rad Laboratories, Inc.). DNA standards for library quantification (Takara Bio Inc., Shiga, Japan) were used as the standard. 10 nM of the cDNA library from every single cell was treated with the NovaSeq 6000 S4 reagent kit and sequenced by Nova-Seq 6000 (151 bp, paired-end) (Illumina KK).

#### Preprocess of scRNA-seq data

The FASTQ quality check of reads was performed using FastQC (version 0.11.5). The reads were trimmed by using the fastq-mcf (version 1.0) with the parameters “-L 150 -l 50 -k 4.0 -q 30”. The trimmed reads were mapped to the human reference genome (GRCh38, primary assembly) using HISAT2 (version 2.1.0) with the parameters “--no-softclip”(Kim et al., 2019). The resulting SAM files were then converted into BAM files using SAMtools (version 1.4). RSeQC (version 1.2) was used for the quality check of the read-mapping(Wang et al., 2012). To mitigate the difference in the number of mapped reads among single cells, the reads in each BAM file were subsampled to 555,421 reads when the BAM file contained more than 555,421 reads using samtools view ‘-s’ option. The featureCounts command in Subread (version 1.6.4) was used to quantify gene-level expression with the parameters “-M -O --fraction -p” and the reference gene model (GENCODE v32, primary assembly)(Liao et al., 2014). Transcript per million (TPM) values were calculated from the gene-level expression quantification result using R (version 3.6.1)(Wagner et al., 2012). R was used for the statistical analyses and figure generation.

#### Pseudotime analysis

The cells with low mapped reads were discarded from the pseudotime analysis. Diffusion maps were performed using the destiny package (version 3.0.1) after retaining genes where at least 3 counts were found in at least 10 cells(Angerer et al., 2016). The pseudotime analysis was performed using the slingshot package (version 1.4.0)(Street et al., 2018) as follows: (1) we selected expressed genes with a count of at least 3 in at least 10 cells. (2) We normalized the count matrix of the expressed genes with the full quantile normalization. (3) We performed diffusion maps on the log-transformed normalized count matrix with the pseudocount of 1 using the “destiny” R package (version 3.0.1). (4) We applied the slingshot analysis on the result of diffusion maps (diffusion components 1 and 2) to estimate the pseudotime. The package gam (version 1.16.1) was used for fitting a generalized additive model with the parameter “family = Gaussian(link = identity)” to search for dynamically regulated genes (from highly variable genes (FDR < 0.01)) and lectins (*q* < 0.05 after the multiple testing correction using the Benjamini & Hochberg method).

#### Seurat analysis

The Seurat package (version_3.9.9.9014) was used to analyze scGR-seq data for hiPSCs and NPCs. The TPM matrix for the scRNA-seq data and the lectin expression matrix for the scGlycan-seq data was used as inputs. The RNA data was preprocessed using the ‘NormalizeData’ function with the default parameters, the ‘FindVariableFeatures’ function with the parameter ‘selection.method = "vst"’, and the ‘ScaleData’ with the default parameters. The lectin data was preprocessed using the ‘NormalizeData’ function with the parameters ‘normalization.method = 'CLR', margin = 2’, the ‘FindVariableFeatures’ function with the default parameters, and the ‘ScaleData’ with the default parameters. Principal component analysis (PCA) was then performed on the RNA data and the lectin data separately using the ‘RunPCA‘ function with the parameter ‘approx=FALSE’. Then, UMAP visualization was computed based on the PCA result of RNA (PC1 to 10) and lectin (PC1 to 20) data individually using the ‘RunUMAP’ function with the default parameters. In parallel, the ‘FindNeighbors’ function with the default parameters was used on the PCA result of RNA (PC1 to 10) and lectin (PC1 to 20) data to define *k* nearest neighbor cells with the parameter ‘resolution = 0.5’. To search differentially expressed genes, ‘FindMarkers’ function with the parameter ‘logfc.threshold=0.25’ was performed and genes which match the criteria, Benjamini-Hochberg adjusted p-value < 0.05, were visualized by ‘DoHeatmap’ function. GO enrichment analysis was performed using the DAVID Bioinformatics Resources 6.8 (https://david.ncifcrf.gov/tools.jsp).

To jointly analyze the RNA and lectin modalities, the weighted nearest neighbor (WNN) workflow in Seurat was performed(Hao et al., 2020). First, to learn cell-specific modality 'weights' and construct a WNN graph that integrates the two modalities, the ‘FindMultiModalNeighbors’ function was used on the PCA results of mRNA (PC1 to 10) and lectin (PC1 to 20) with the parameters ‘knn.range = 50, k.nn=10’. Then, UMAP visualization was computed based on a weighted combination of RNA and lectin data using the ‘RunUMAP’ function with the default parameters. In parallel, graph-based clustering was performed based on the WNN graph using the ‘FindClusters’ function with the parameter ‘resolution = 0.2’. To evaluate the similarity of the clustering with the cell type annotation, the adjusted Rand index was calculated using the ‘mclust’ R package (version 5.4.6).

#### PLS regression of scRNA-seq and scGlycan-seq data

The package ropls (version 1.18.8) was used for PLS regression with scRNA-seq and scGlycan-seq data (Thevenot et al., 2015). Let X be n×m gene expression matrix (scRNA-seq) and Y be n×p lectin matrix (scGlycan-seq), where *n* is the number of cells, *m* is the number of genes, and *p* is the number of lectins. PLS regression decomposes X and Y to maximize the covariance between T and U as follows:

$$X=TP^{⊤}+E$$

$$Y=UQ^{⊤}+F$$

where T and U are n×l matrices which is the projection of X and Y, respectively, and P and Q are m×l andp×l orthogonal loading matrices, respectively, and matrices E and F are the error terms. The l components are analogous to principal components. The associations of genes and lectins were calculated as $PQ^{⊤}$ (Figures 8 and S14). The glycogene annotation was retrieved from GlycoGene Database (GGDB, https://acgg.asia/ggdb2/) (Narimatsu, 2004).

#### Flow cytometry

Lectins were recombinantly expressed in *Escherichia coli* and purified by affinity chromatography as described(Tateno, 2020; Tateno et al., 2011). Lectins were labeled with R-phycoerythrin (PE) using the R-phycoerythrin Labeling Kit (Dojindo Laboratories Co. Ltd., Kumamoto, Japan). 1x10^5^ cells were suspended in 100 μl of PBS/BSA and incubated with 10 μg ml^-1^ of PE-labeled lectins on ice for 1 h. For intracellular staining, cells were fixed with 4% paraformaldehyde at room temperature for 10 min and permeabilized with 0.1% saponin in PBS at room temperature for 10 min. Cells were then incubated with anti-PAX6 mAb (x20, clone No. O18-1330, BD Biosciences, CA, USA) on ice for 1 h. Flow cytometry data were acquired on a CytoFLEX (Beckman Coulter, Inc., CA ) and analyzed using the FlowJo software (FlowJo, LLC., OR).

#### Fluorescence staining

Cells were fixed with 4% paraformaldehyde at room temperature for 20 min. After blocking with PBS containing 1% BSA and 0.2% Triton-X at room temperature for 30 min, the cells were stained with anti-Nestin mAb (71.1 μg/ml, Clone 25/NESTIN (RUO), BD Biosciences), anti-PAX6 mAb (82.1 μg/ml, clone No. O18-1330, BD Biosciences) or 1 mg/mL of Cy3-labeled lectins at room temperature for 60–90 min. Thereafter, the cells were observed by fluorescence microscopy (IX51, Olympus, Tokyo, Japan). The nucleus was stained with Hoechst 33342 (x 1,000, Dojindo Laboratories).

#### Quantitative RT-PCR (qRT-PCR)

Total RNA was extracted using RNeasy Mini Kit (QIAGEN, Hilden, Germany) according to the manufacturer’s protocol. Equal amounts of total RNA from each samples were then reverse-transcribed into cDNA using QuantiTect Reverse Transcription Kit (QIAGEN). qRT-PCR was performed using PowerUp SYBR Green Master Mix (Thermo Fisher Scientific) and CFX Connect (Bio-Rad Laboratories, Inc.). The mRNA expressions of indicated genes were normalized to that of the GAPDH gene. The following primers were used for qRT-PCR: GAPDH-fw(forward): GGCTGGCATTGCCCTCAACG; GAPDH-rv(reverse): AGGGACTCCCCAGCAGTGAG; OCT4-fw: GGAAGGAATTGGGAACACAAAGG; OCT4-rv: AACTTCACCTTCCCTCCAACCA; SOX1-fw: GTCCATCTTTGCTTGGGAAA; SOX1-rv: TAGCCAGGTTGCGAAGAACT; NESTIN-fw: CAGCGTTGGAACAGAGGTTGG; NESTIN-rv: TGGCACAGGTGTCTCAAGGGTAG; PAX6-fw: GTCCATCTTTGCTTGGGAAA; PAX6-rv: TAGCCAGGTTGCGAAGAACT; FOXG1-fw: GCGCAAATGCCGCATAAAT; FOXG1-rv: AAACACGGGCATATGACCACAG.

### Quantification and statistical analysis

The data are shown as average ± standard division or violin plot. The statistical analyses were performed with paired t test, Benjamini & Hochberg method or Brunner-Munzel test using R version 3.6.1. Statistical significance was set at *p*-value < 0.05.

## Data Availability

All raw data of Glycan-seq are provided in supplementary tables. All raw data of scRNA-seq have been deposited to the Gene Expression Omnibus: GSE151642. The code of barcode DNA counting system is available from the lead contact upon request. Any additional information required to reanalyze the data reported in this paper is available from the lead contact upon request.
