# Supplementary material for: Integrated analysis of glycan and RNA in single cells
Source: iScience. 2021 Jul 17;24(8):102882. doi: 10.1016/j.isci.2021.102882 (PMC8349903; doi:10.1016/j.isci.2021.102882)
Supplement: Document S1. Figures S1–S24 [file mmc1.pdf]

iScience, Volume *24*

Supplemental information

Integrated analysis of glycan and RNA in single cells

Fumi Minoshima, Haruka Ozaki, Haruki Odaka, and Hiroaki Tateno

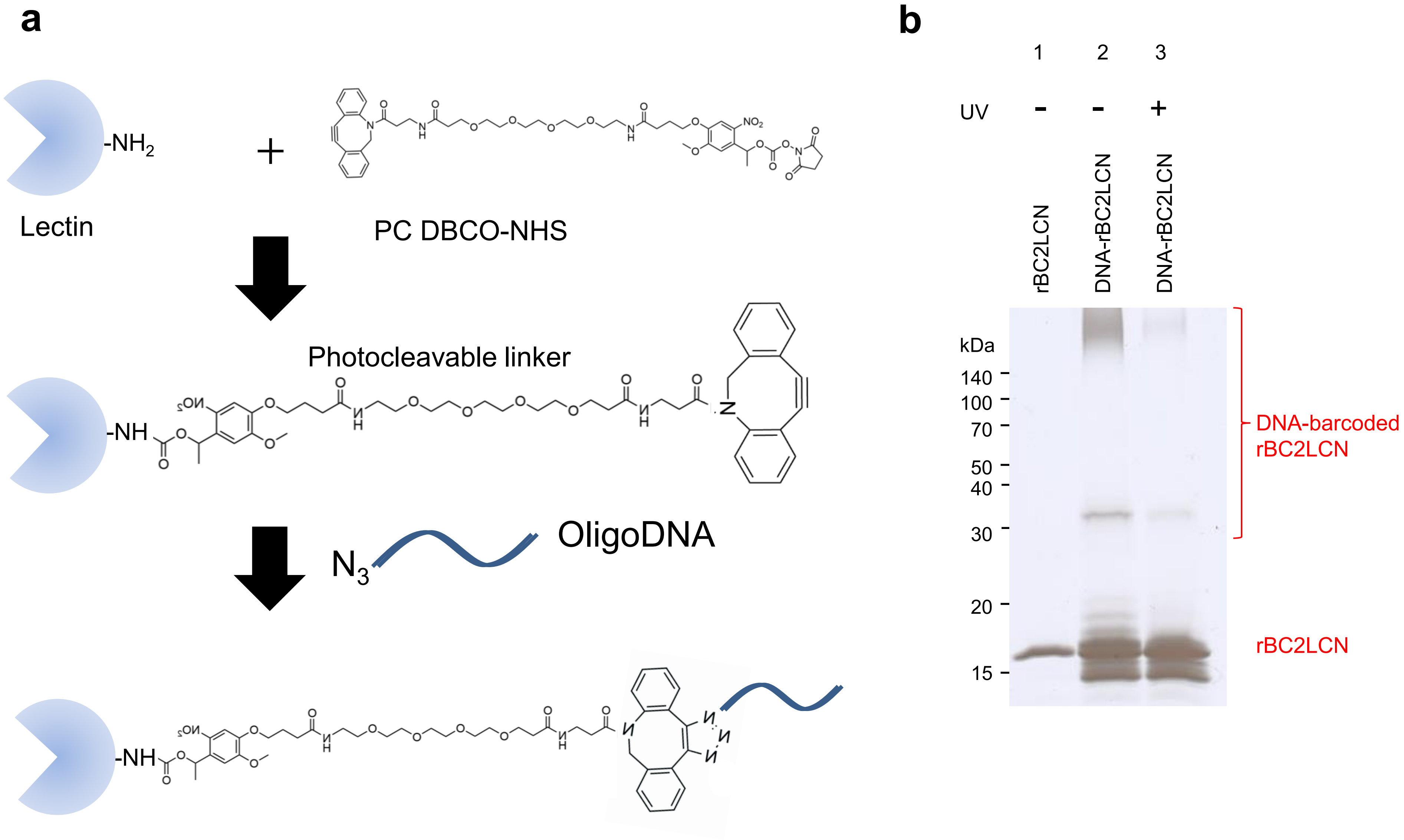

Figure S1. Conjugation of lectins to DNA oligonucleotides, Related to Figure 1. **(a)** Illustration of the protocol of the conjugation of lectins with DNA oligonucleotides. **(b)** rBC2LCN shows a single band at 16 kDa (lane 1). DNA-barcoded rBC2LCN exhibited a high-molecular weight smear band at >140 kDa (lane 2). Cleavage of DNA barcodes from rBC2LCN by UV exposure collapses the smear to the MW of rBC2LCN (16 kDa) (lane 3).

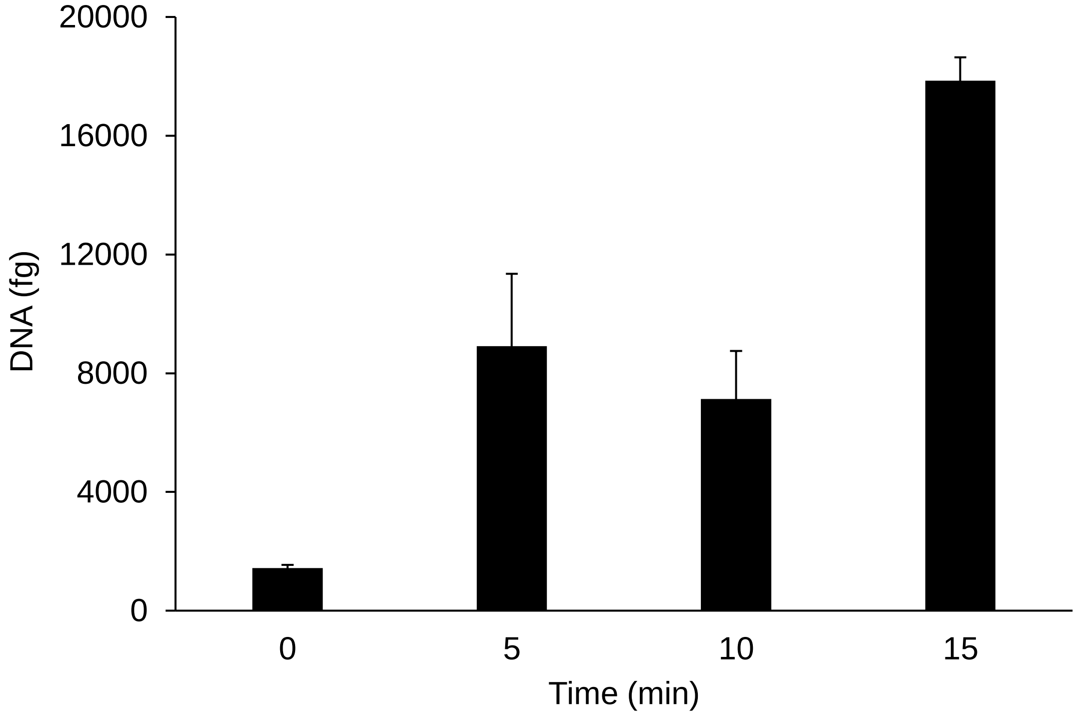

Figure S2. Optimization of UV exposure time to release DNA barcode from DNA-barcoded lectins, Related to Figure 1. Capan-1 was incubated with 1 μg/mL of DNA-barcoded rBC2LCN on ice for 1 h. UV light was exposed to cells for 0, 5, 10, 15 min and the released DNA was recovered and analyzed by qRT-PCR.

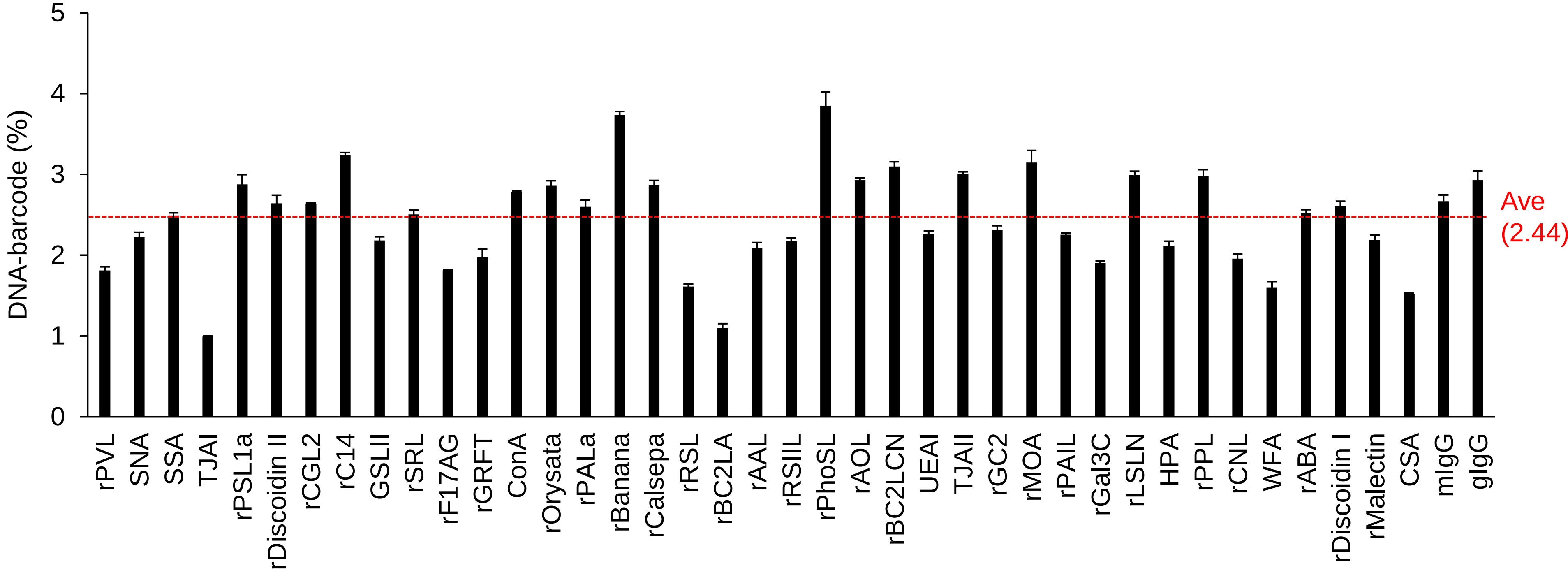

Figure S3. PCR amplification bias of DNA barcodes, Related to Figure 1. DNA barcode (1 pM) of 41 probes (lectins and antibodies) was mixed and amplified using NEBNext UltraII, and i5-index and i7-index primers. The PCR products were then purified by the Agencourt AMPure XP kit, followed by the manufacturer's protocol. The size and the quantity of the PCR products were analyzed by MultiNA. The PCR products (4 nM for each DNA barcode) were treated with the Miseq Reagent Kit v2 50 Cycles and sequenced by the MiSeq sequencer (26 bp, paired-end). DNA barcodes were directly extracted from the reads in the FASTQ format. The number of DNA barcodes was counted using the developed software, Barcode DNA counting system. Each DNA barcode count was divided by the total lectin barcode count and expressed as a percentage (%) for each lectin.

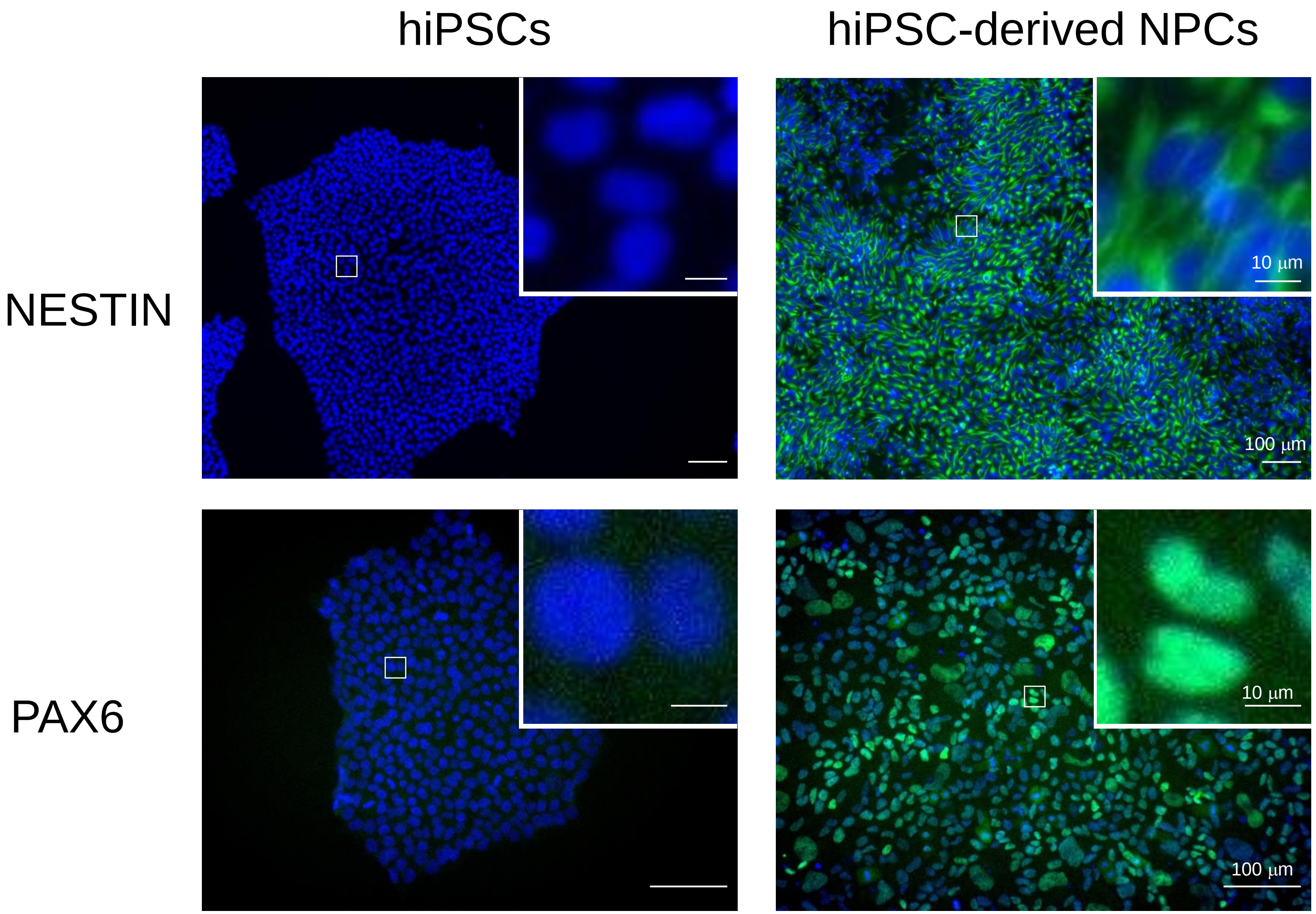

Green: antibody staining. *Blue*: nuclear staining.

Figure S4. Fluorescence staining of hiPSCs and hiPSCs-derived NPCs by anti-NESTIN or PAX6 mAb, Related to Figure 4. hiPSCs and hiPSC-derived hNPCs (Nestin: 17-day differentiation, PAX6: 25-day differentiation) were fixed with 4% paraformaldehyde at room temperature for 20 min. After blocking with PBS containing 1%BSA and 0.2% Triton-X at room temperature for 30 min, cells were stained with anti-Nestin mAb (71.1 µg/ml) or anti-PAX6 mAb (82.1 µg/ml) and Hoechst33342 (1 µg/ml) at room temperature for 90 min. Insets show high magnification of selected fields. Nuclear: *blue*. Nestin and PAX6: *green*. Scale bar: 100 µm or 10 µm (Insets).

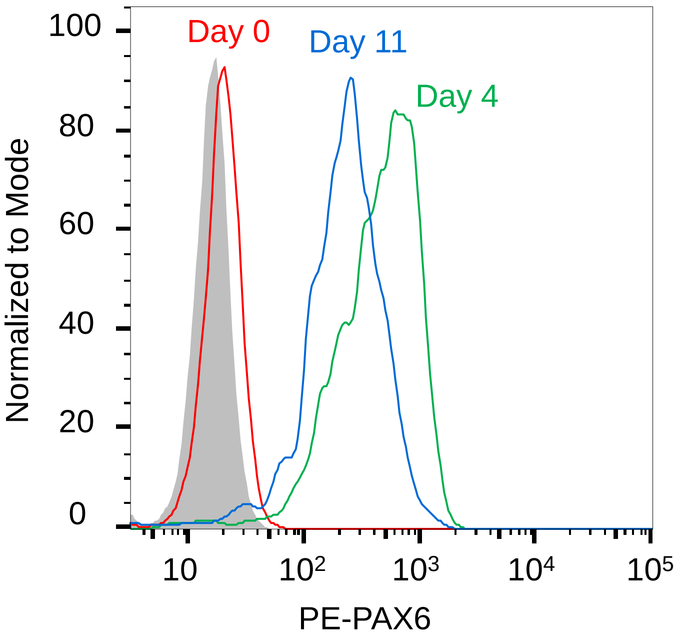

Figure S5. Flow cytometry analysis of hiPSCs during differentiation into NPCs, Related to Figure 4. hiPSCs before (Day 0, *red line*) and after differentiation into neural progenitor cells (Day 4, *green line*; Day 11, *blue line*) were fixed with 4% paraformaldehyde at room temperature for 10 min and permeabilized with PBS containing 0.1% saponin at room temperature for 10 min. Cells were stained with PE-labeled anti-PAX6 mAb (clone No. O18-1330) on ice for 1 h and analyzed by flow cytometry.

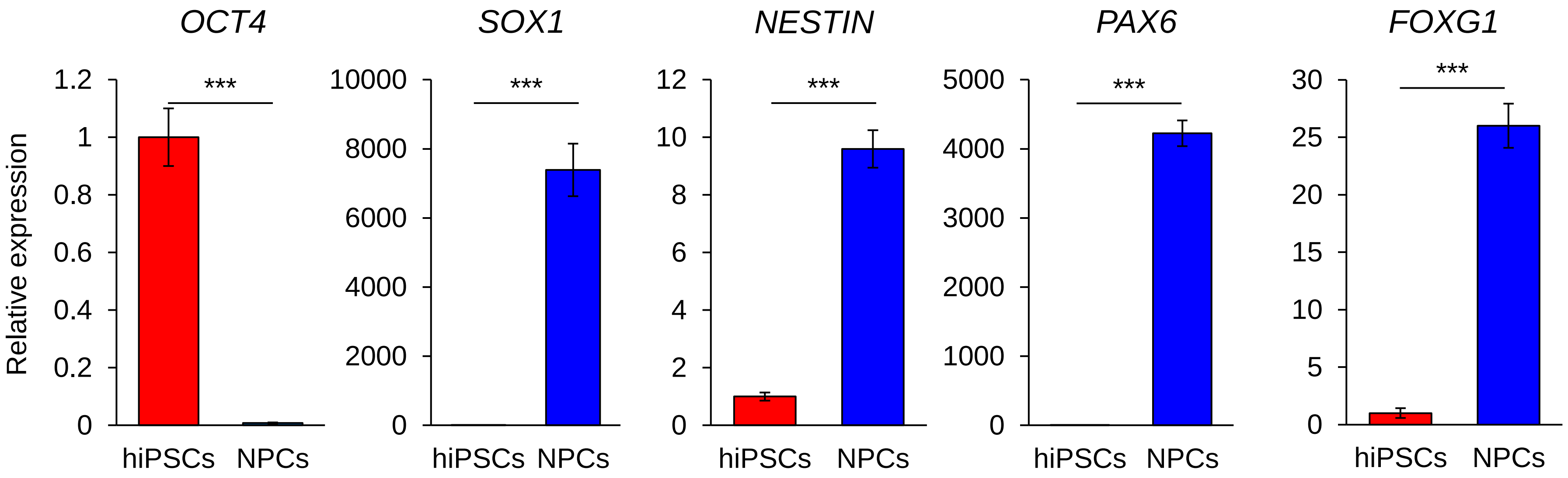

Figure S6. Relative expression of mRNA of hPSC (OCT4) and NPC markers (SOX1, NESTIN, PAX6, FOXG1) in hiPSCs and hiPSC-derived hNPCs, Related to Figure 4. The expression of mRNA was measured by qRT-PCR. Data are shown as relative to hiPSC data. Data are shown as average ± SD of triplicate experiments. *** $p < 0.001$. *t-test.*

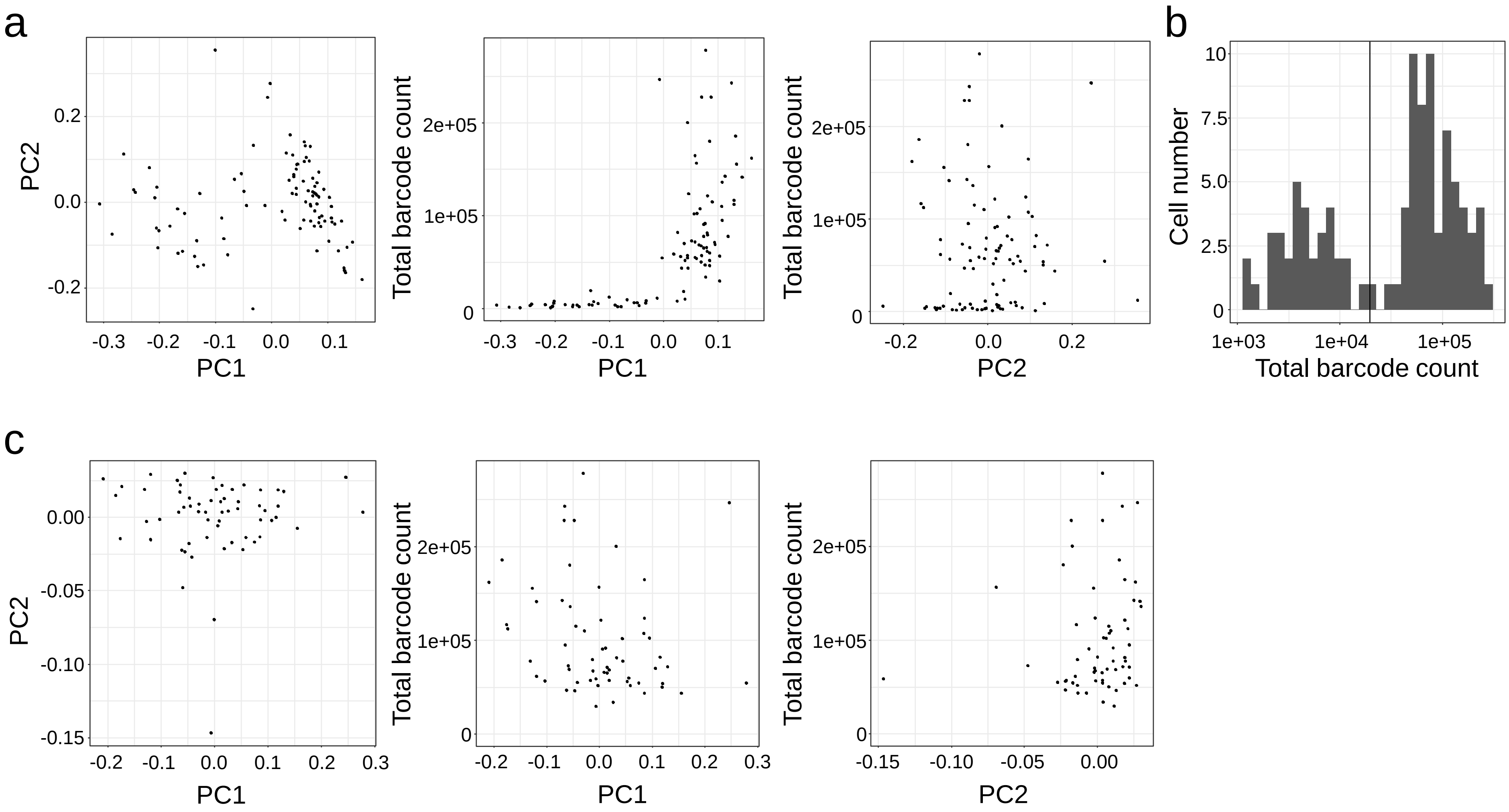

Figure S7. Quality control of scGlycan-seq data, Related to Figure 5. **(a)** PCA plot of scGlycan-seq data of fibroblasts (n = 96) (*left*). The relationship of PC1 or PC2 with total barcode counts was analyzed (*middle*, *right*). **(b)** Histogram of total barcode count of fibroblasts. Black line indicates threshold determined by Otshu’s method (barcode count > 19465). **(c)** PCA plot of scGlycan-seq data of fibroblasts after removal of cells with low total barcode counts (*left*). The number of samples was reduced from 96 to 61. The relationship of PC1 or PC2 with total barcode counts was illustrated (*middle*, *right*). No obvious bias was observed in these data sets.

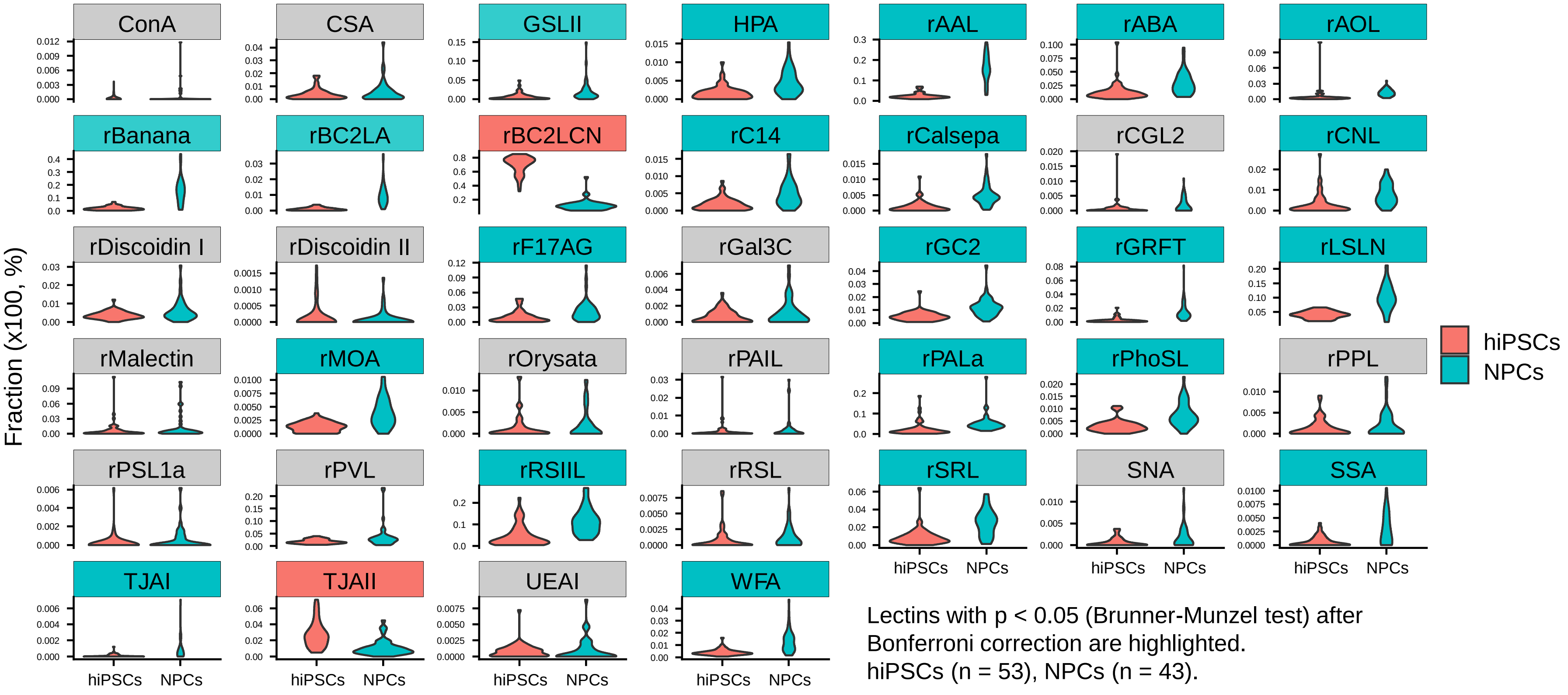

Figure S8. scGlycan-seq data of hiPSCs and NPCs, Related to Figure 5. Violin plots showing the signal level of each lectin in scGlycan-seq data of hiPSCs (n = 53), NPCs (n = 43). Lectins with p < 0.05 (Bunner-Munzel test) after Bonferoni correction are highlighted in red and cyan when the average signal was higher in hiPCSs and NPCs, respectively.

a

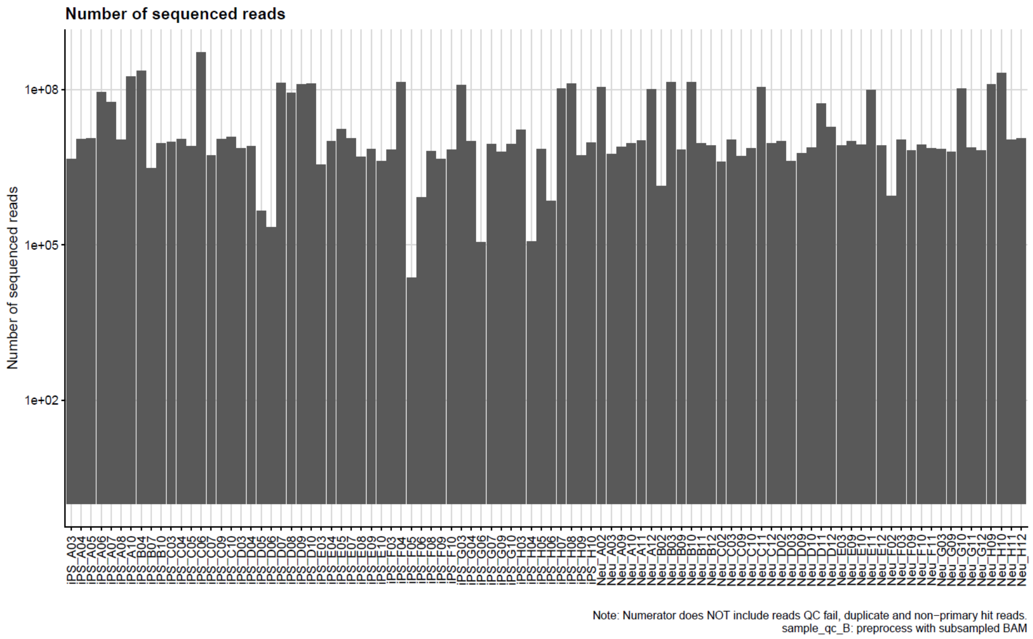

b

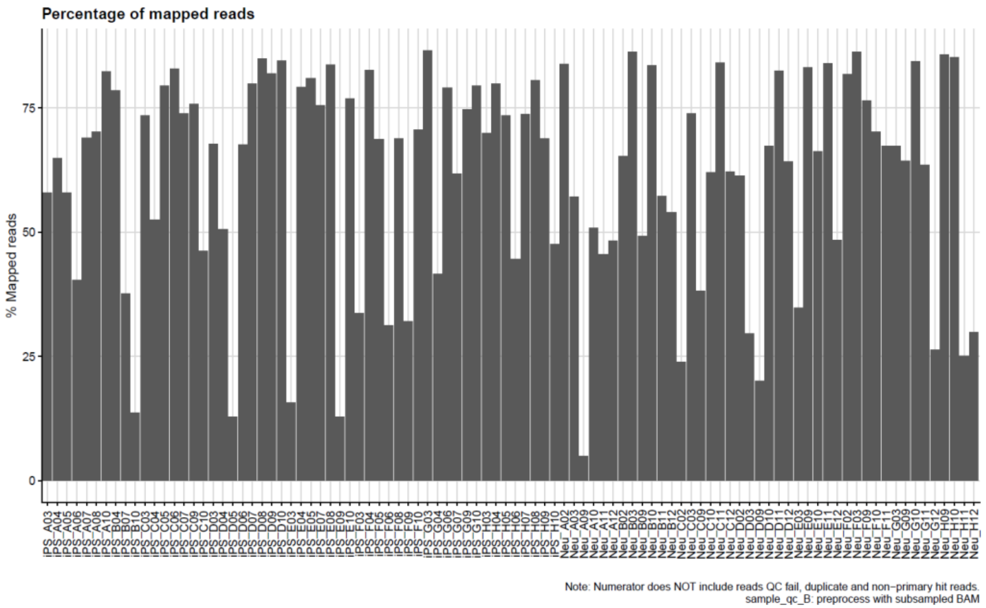

Figure S9. Quality control of scRNA-seq data, Related to Figure 7. **(a)** The number of total sequenced reads after the read trimming of FASTQ reads. **(b)** The percentage of reads mapped to the human genome.

a

b

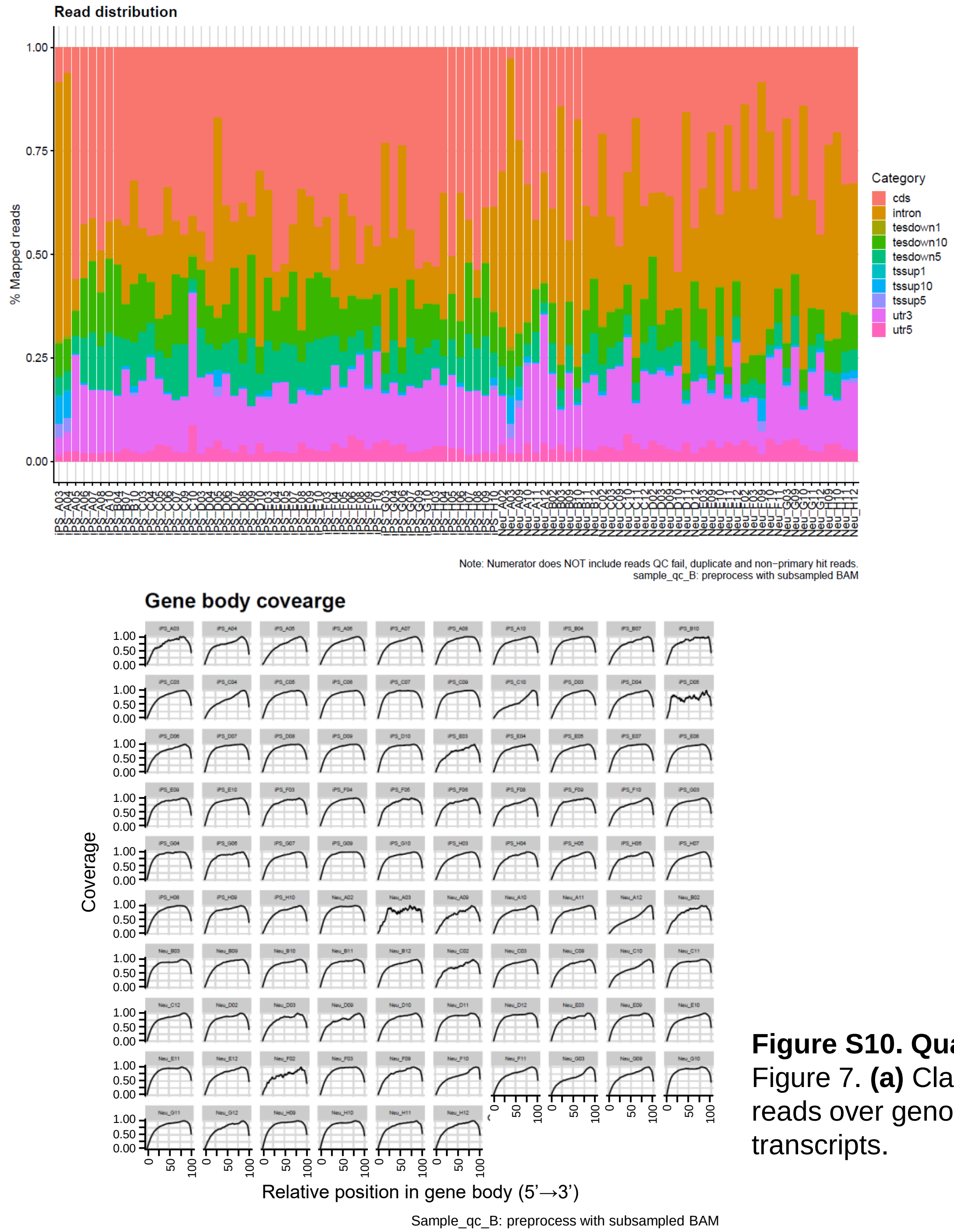

Figure S10. Quality control of scRNA-seq data, Related to Figure 7. **(a)** Classification and distribution of uniquely mapped reads over genomic features. **(b)** Mean read coverage over transcripts.

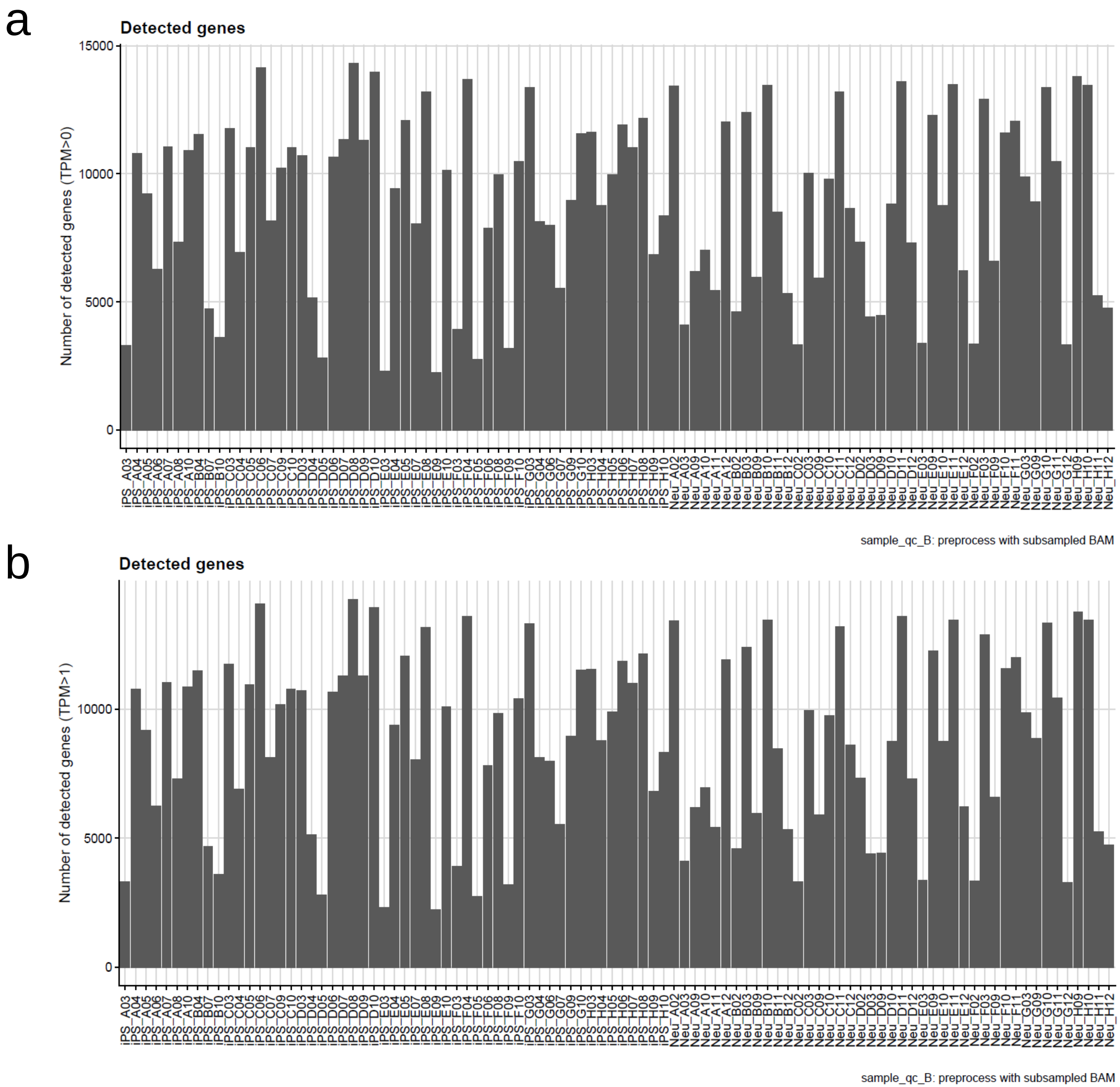

Figure S11. Quality control of scRNA-seq data, Related to Figure 7. **(a)** The number of detected genes with TPM (transcript per million) > 0. **(b)** The number of detected genes with TPM (transcript per million) > 1.

a

b

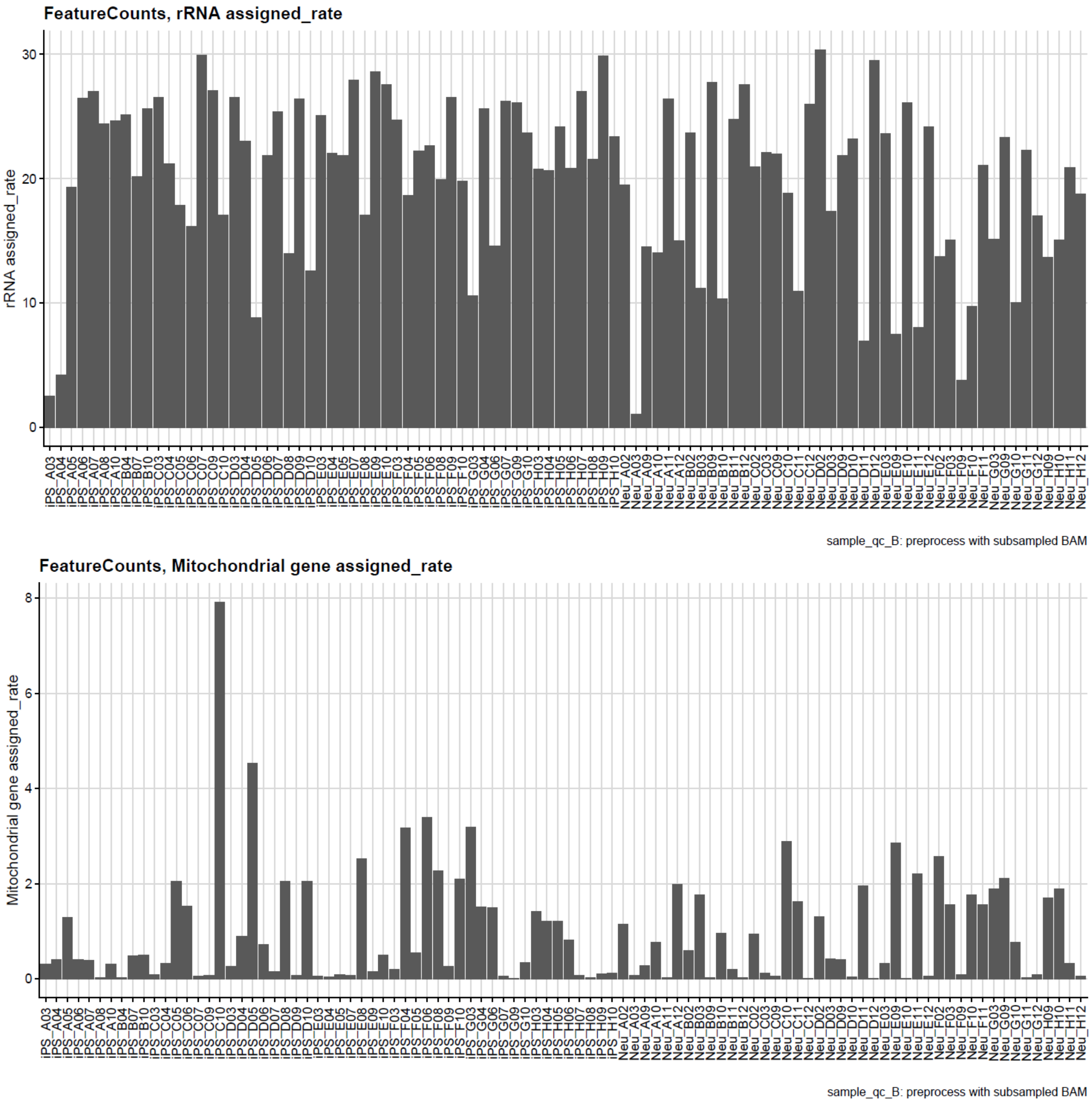

Figure S12. Quality control of scRNA-seq data, Related to Figure 7. (a) The percentage of mapped reads that were overlapped with rRNA gene annotations on the genome. (b) The percentage of mapped reads that were overlapped with mitochondrial rRNA gene annotations on the genome.

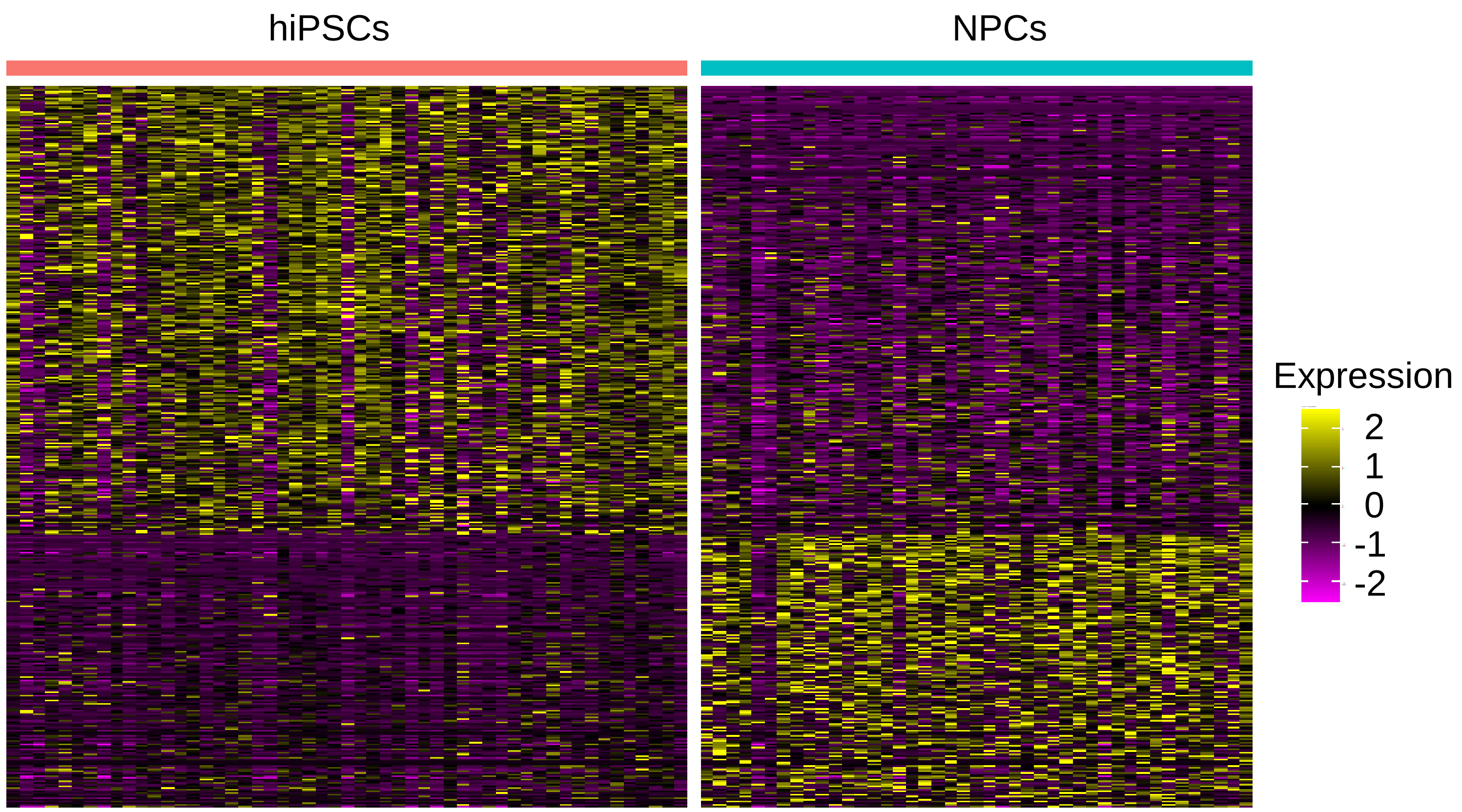

Figure S13. scRNA-seq analysis of differentially expressed genes between hiPSCs and NPCs, Related to Figure 7. Heatmap of differentially expressed genes (DEGs) between iPSCs and NPCs. Criteria for DEG selection was set at log2(FoldChange) > 0.25 and Benjamini-Hochberg adjusted $p < 0.05$ (Mann-Whitney U test). Whole DEG lists were included in Supplementary Table 10.

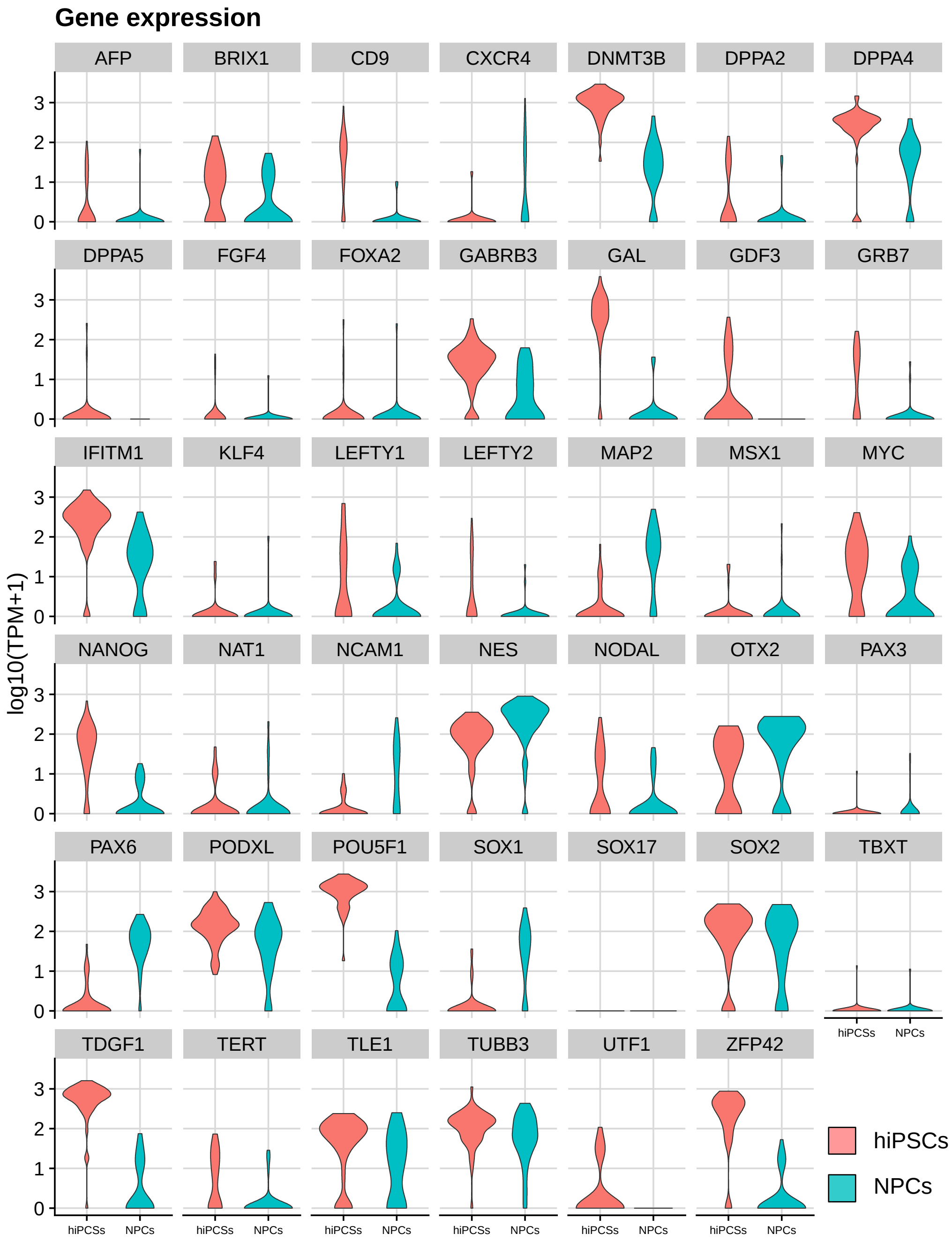

Figure S14. Gene expression of 41 selected markers by scRNA-seq, Related to Figure 7. Violin plots show gene expression levels of 41 selected markers for hiPSCs (n = 53, *red*) and NPCs (n = 43, *cyan*) measured by scRNA-seq. Each point represents a single cell. The y-axes represent the log10(TPM+1).

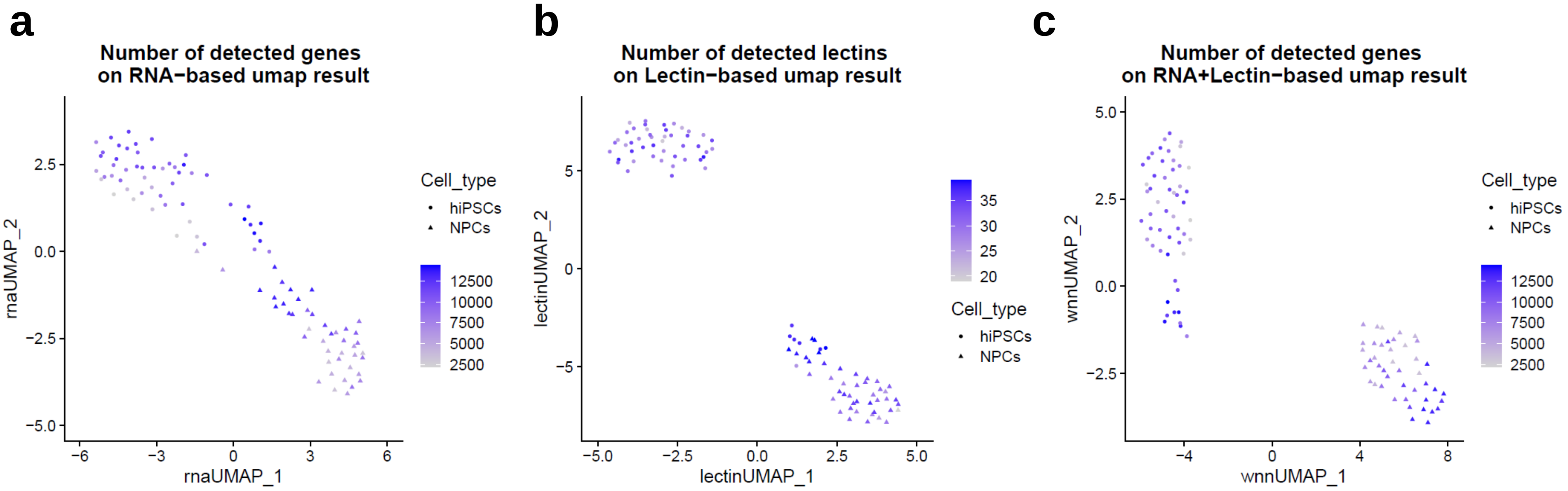

Figure S15. The number of detected genes or lectins on UMAP visualization, Related to Figure 7. The number of detected genes or lectins are shown on UMAP visualization based on (a) only the scRNA-seq data and (b) only the scGlycan-seq, (c) both scRNA-seq and scGR-seq. Color represents the number of detected genes (a and c) or lectins (b). Circles and triangles represent hiPSCs (n = 53) and NPCs (n = 43), respectively.

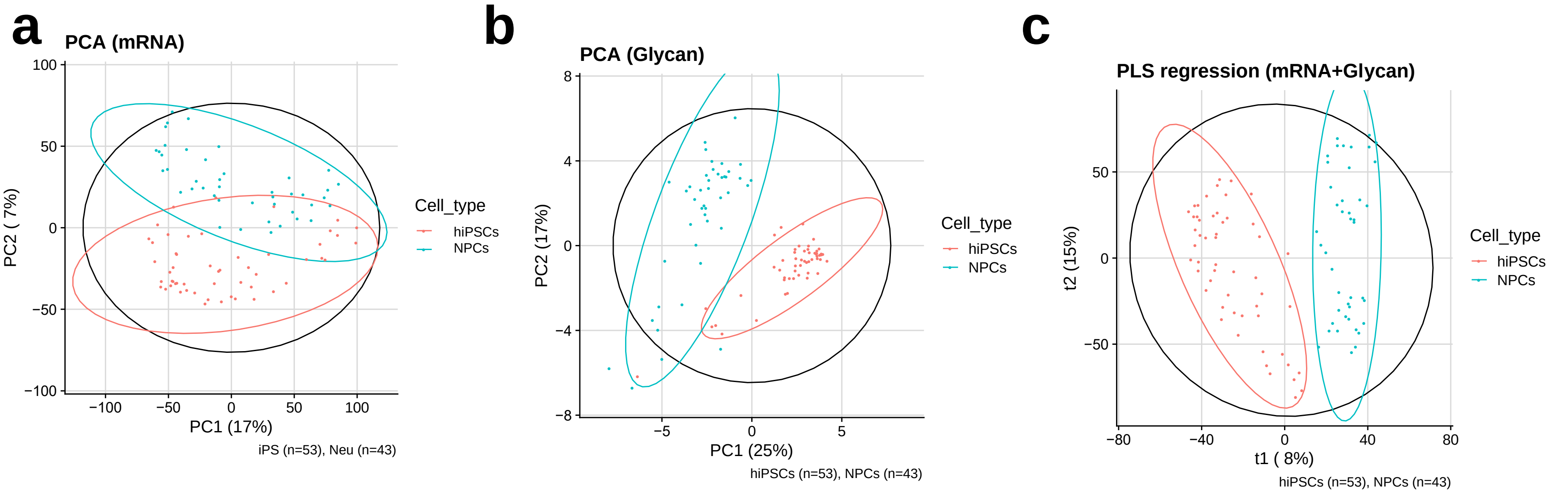

Figure S16. PCA and PLS regression of scGR-seq, Related to Figure 7. **(a)** PCA of scRNA-seq data of hiPSCs (n = 53, blue) and hiPSC-derived NPCs (n = 43, *red*). The x-axis and y-axis represent the first and second principal components (PC1 and PC2), respectively, and the percentages in the parenthesis indicate the proportions of explained variance. **(b)** PCA of scGlycan-seq data of hiPSCs (n = 53, *blue*) and hiPSC-derived NPCs (n = 43, red). The x-axis and y-axis represent the first and second principal components (PC1 and PC2), respectively, and the percentages in the parenthesis indicate the proportions of explained variance. **(c)** Partial least squares (PLS) regression analysis of scRNA-seq and scGlycan-seq data of hiPSCs (n = 53, blue) and hiPSC-derived NPCs (n = 43, red). The x-axis and y-axis represent the first and second components, respectively. Unlike PCA, PLS regression utilizes information from both scRNA-seq and scGlycan-seq data in the construction of components. scGlycan-seq data are available in Supplementary Tables 6, 7, 8, and 9.

p2

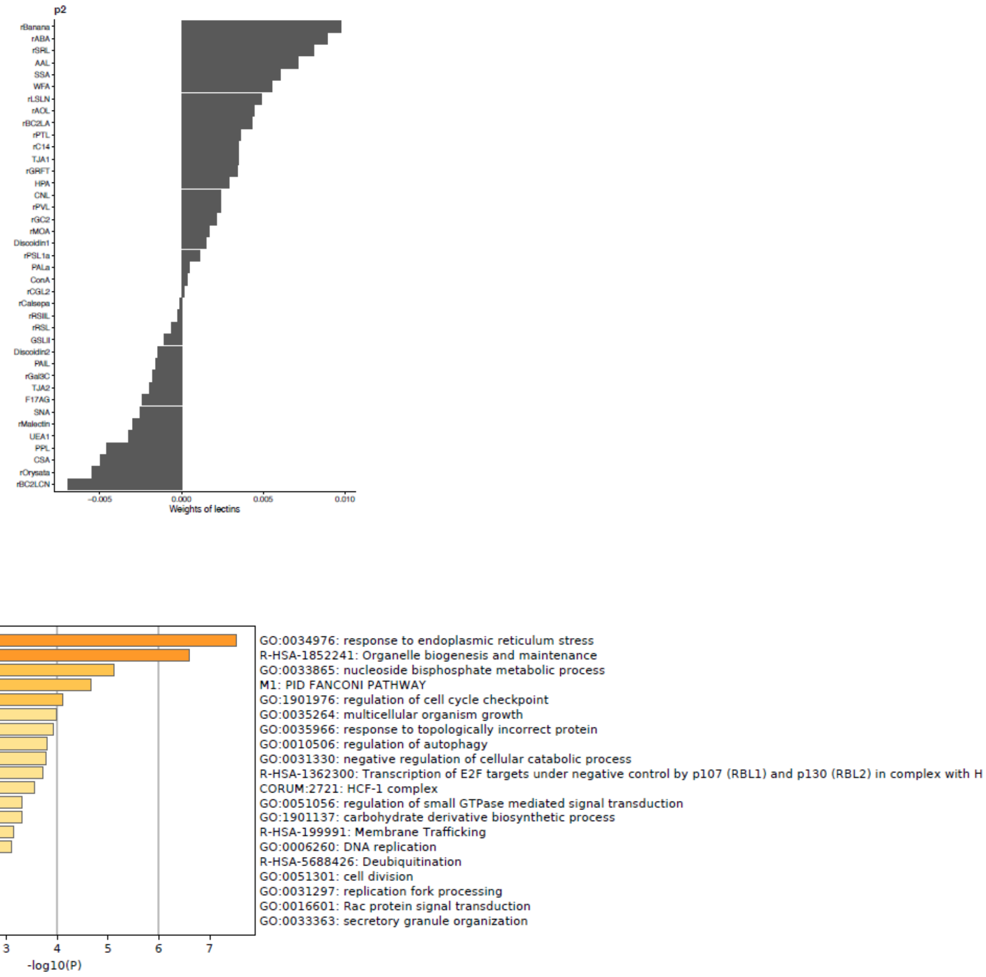

Figure S17. PLS regression, Related to Figure 8. Weights of lectins for the component (p2) from the PLS regression results. Enriched gene sets for the genes associated with the component (p2) of the PLS regression results. Gene enrichment analysis was performed with Metascape (*bottom*). The results of gene enrichment analysis are available in Table S12.

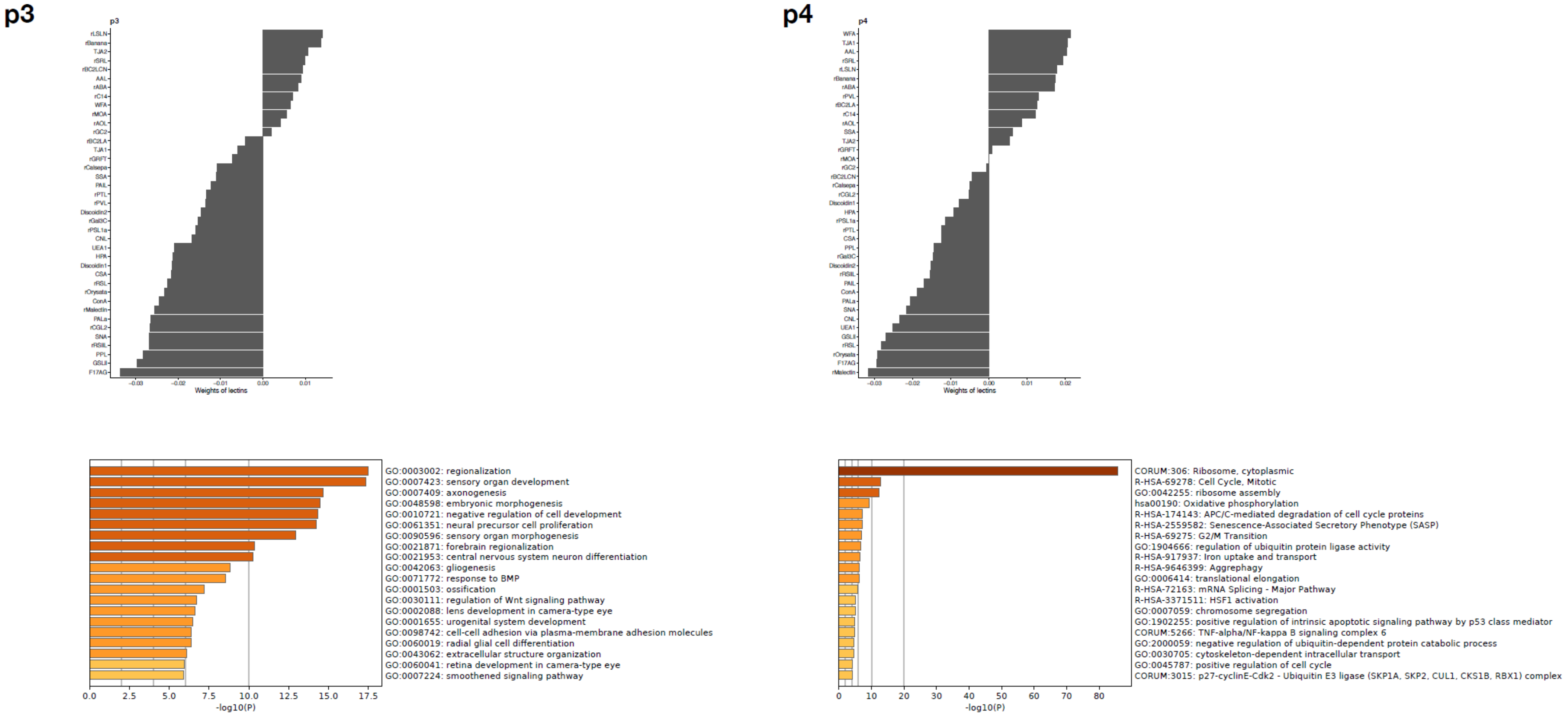

Figure S18. PLS regression, Related to Figure 8. Weights of lectins for the component (p3 and p4) from the PLS regression results. Enriched gene sets for the genes associated with the component (p3 and p4) of the PLS regression results. Gene enrichment analysis was performed with Metascape (*bottom*).

p5

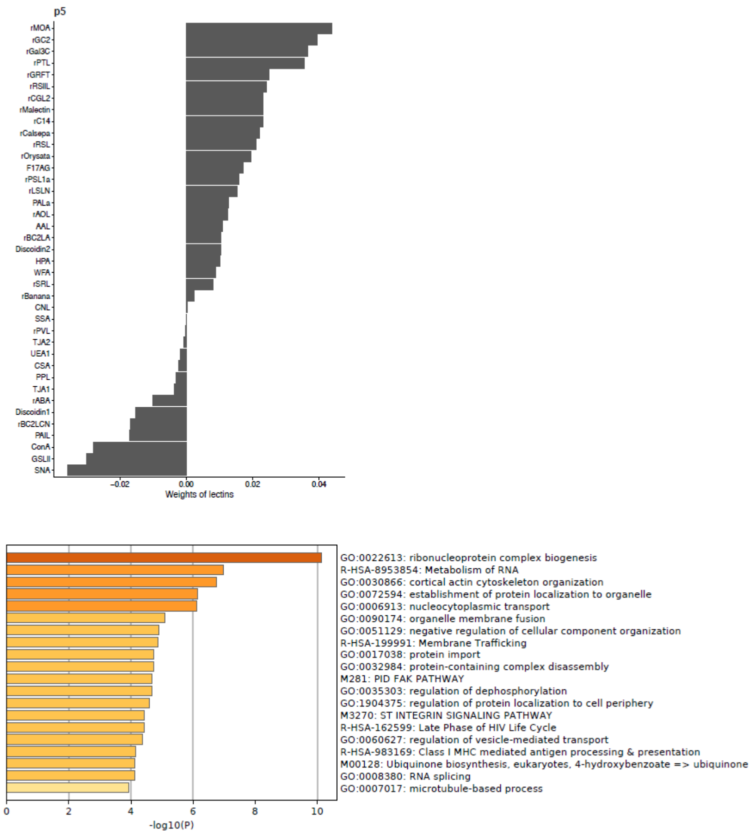

p6

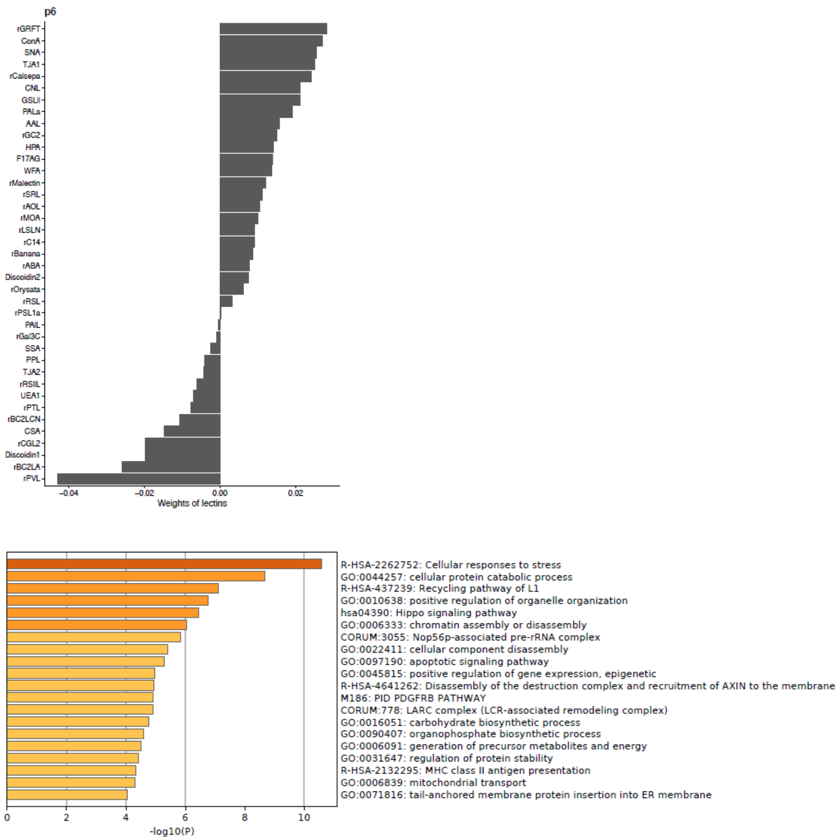

Figure S19. PLS regression, Related to Figure 8. Weights of lectins for the component (p5 and p6) from the PLS regression results. Enriched gene sets for the genes associated with the component (p5 and p6) of the PLS regression results. Gene enrichment analysis was performed with Metascape (*bottom*).

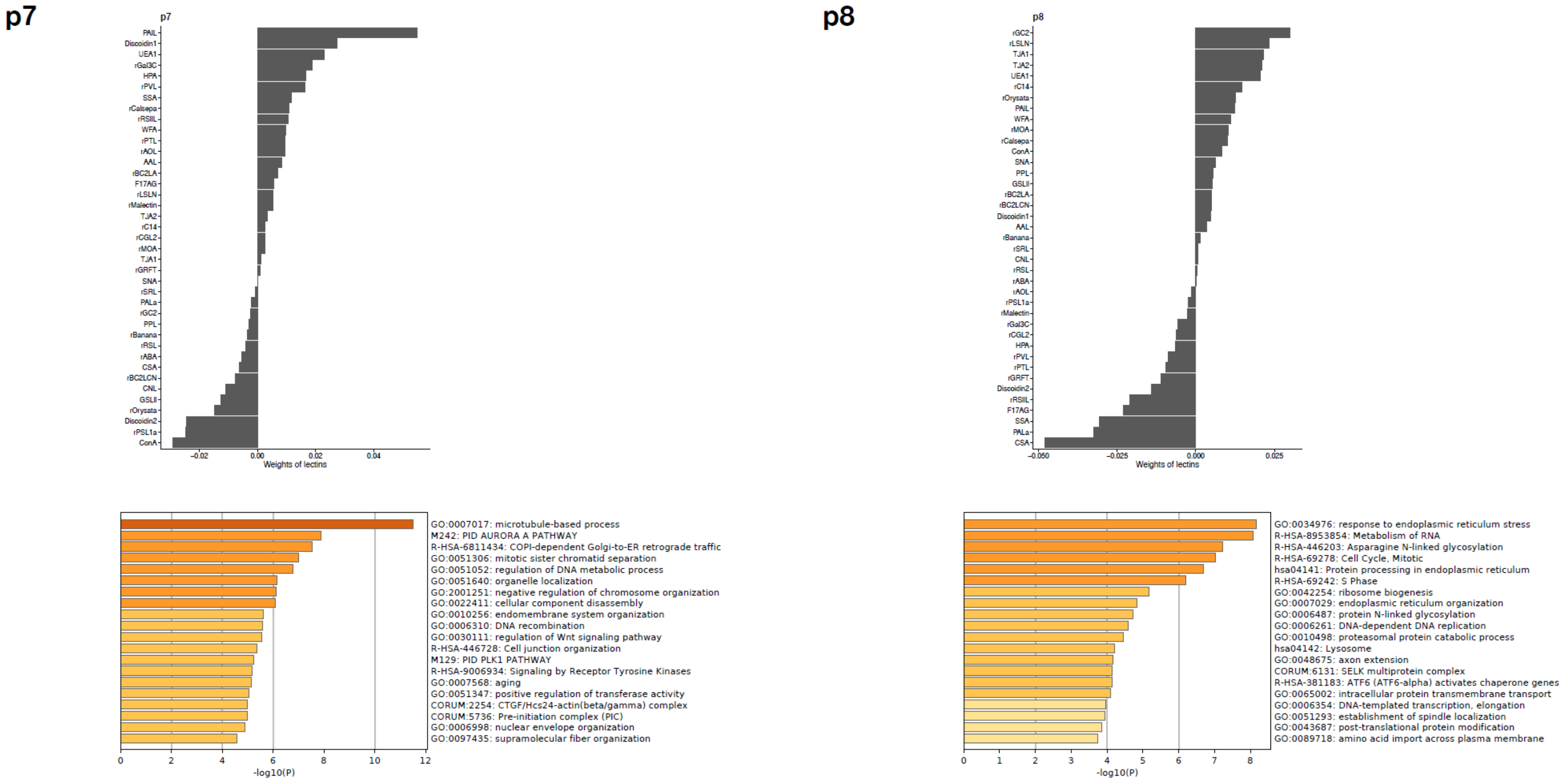

Figure S20. PLS regression, Related to Figure 8. Weights of lectins for the component (p7 and p8) from the PLS regression results. Enriched gene sets for the genes associated with the component (p7 and p8) of the PLS regression results. Gene enrichment analysis was performed with Metascape (*bottom*).

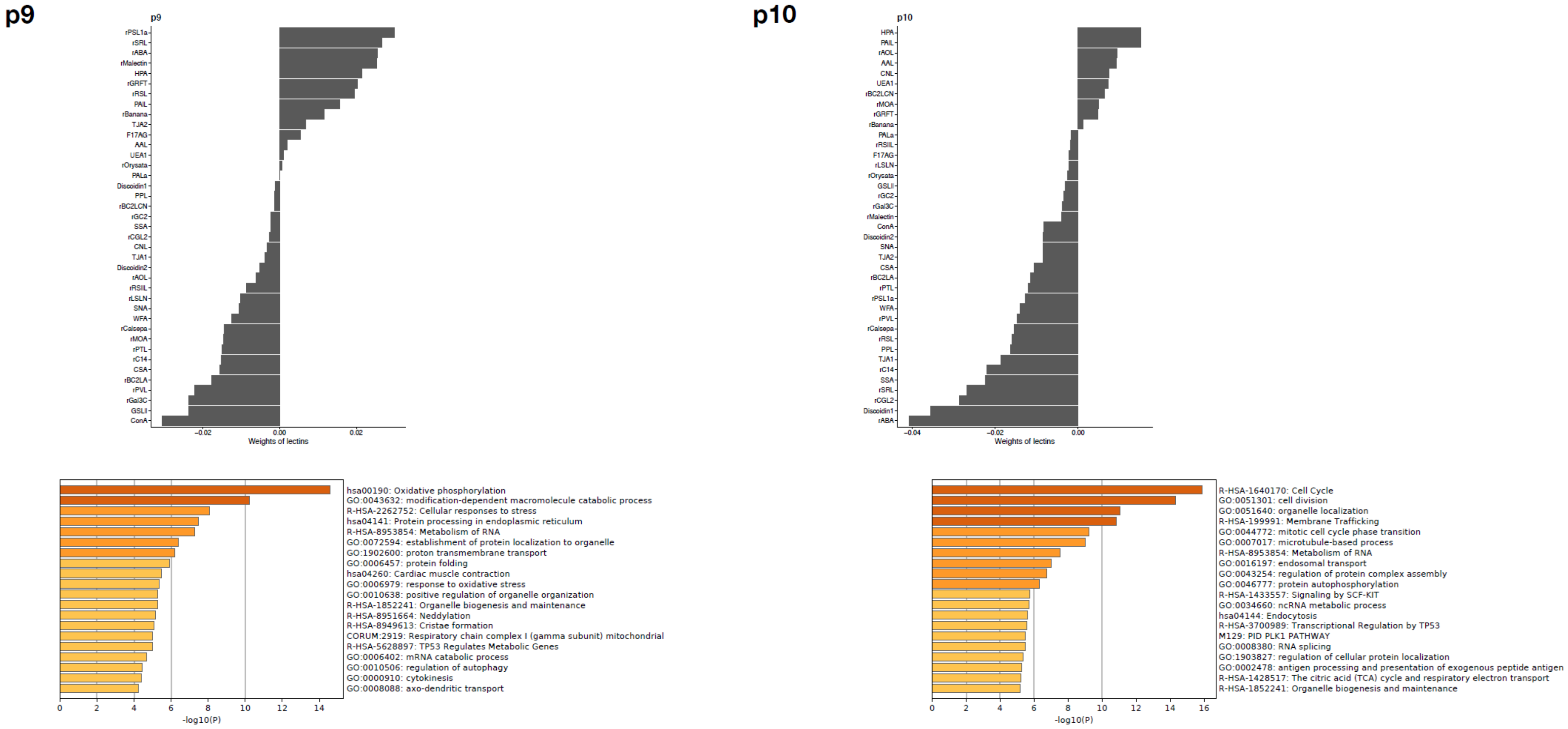

Figure S21. PLS regression, Related to Figure 8. Weights of lectins for the component (p9 and p10) from the PLS regression results. Enriched gene sets for the genes associated with the component (p9 and p10) of the PLS regression results. Gene enrichment analysis was performed with Metascape (*bottom*).

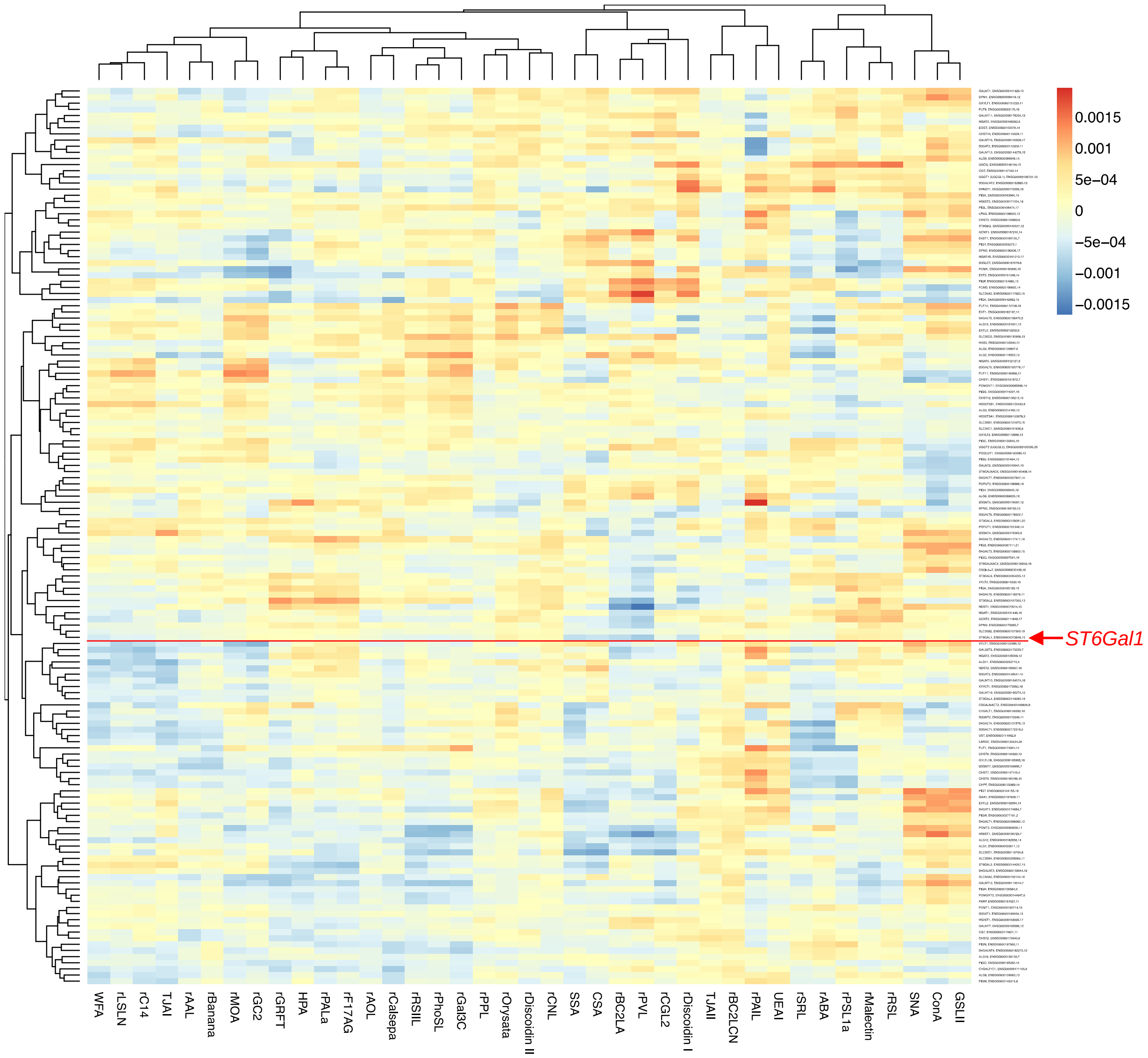

Figure S22. Correlation between lectin signal and glycosyltransferase gene, Related to Figure 8. A heat map shows the association of glycogenes and lectins inferred by PLS regression. For each pair of a gene and a lection, the association was calculated by multiplying the gene loading and lectin loading for each component and summing the products across components. Rows represent genes and columns represent lectins.

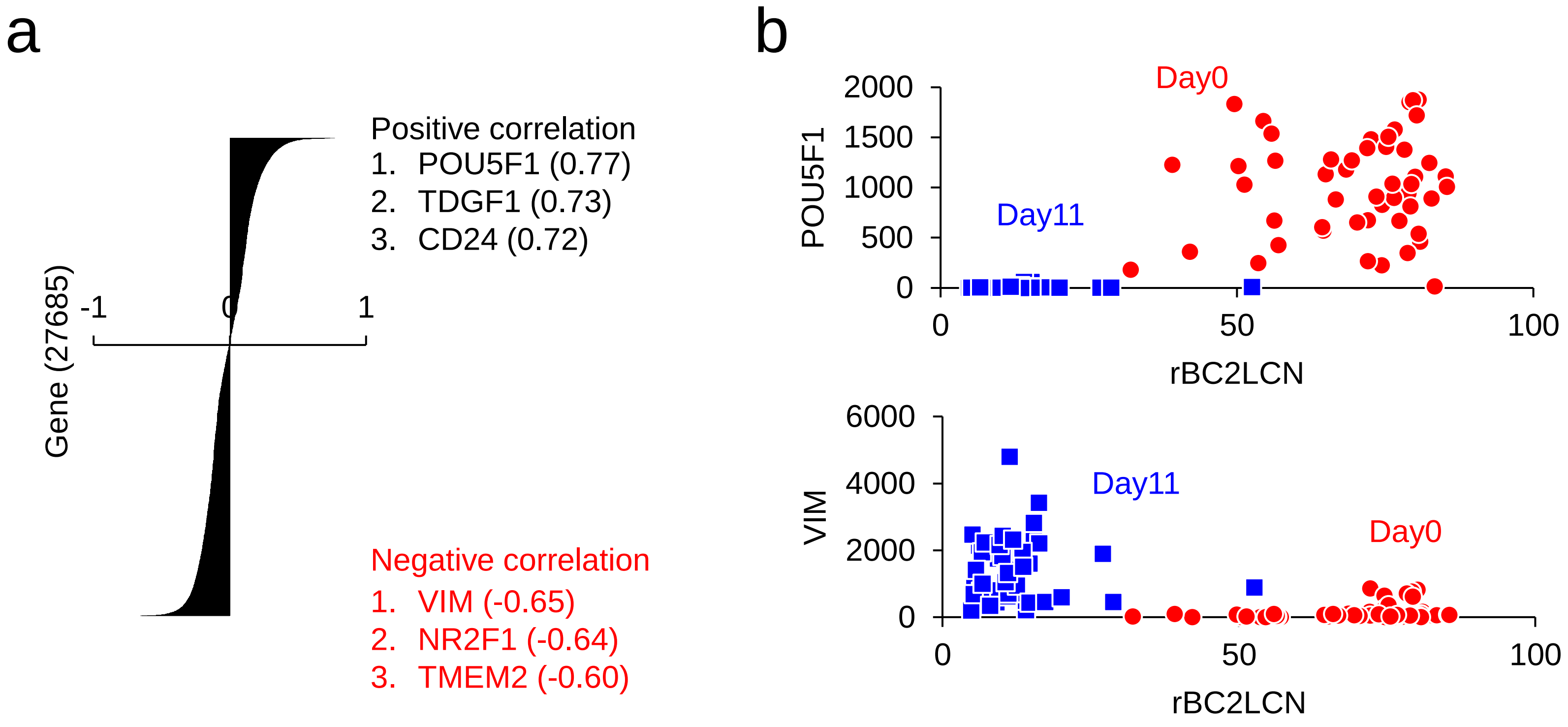

Figure S23. Correlation of genes with rBC2LCN in hiPSC and NPCs, Related to Figure 9. **(a)** rBC2LCN showed the highest positive correlation with hPSC marker POU5F1 and the highest positive correlation with NPC marker VIM. **(b)** Correlation between rBC2LCN with *POU5F1* (*top panel*) and *VIM* (*bottom panel*).

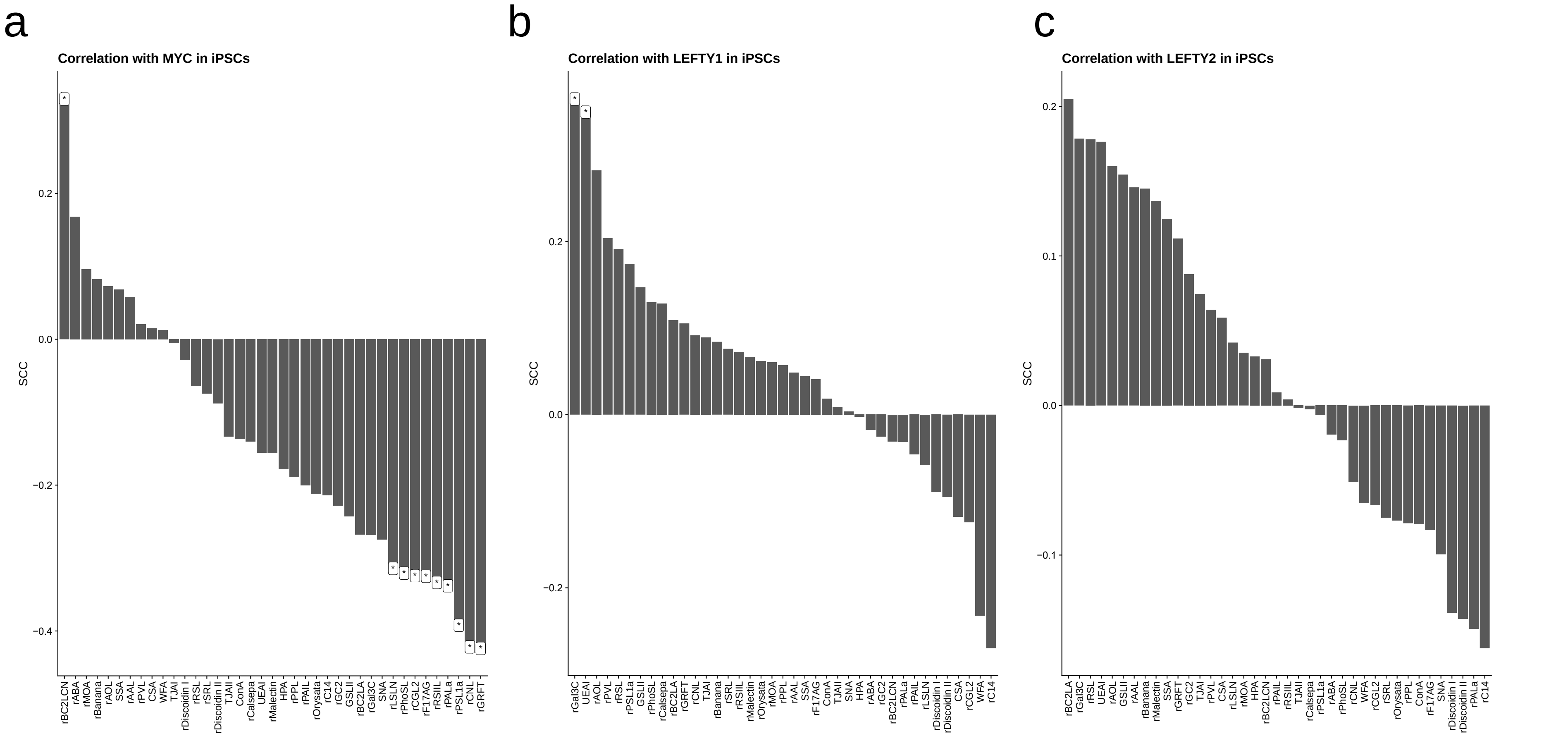

Figure S24. Correlation of lectins with fluctuating genes in hiPSCs, Related to Figure 9. The Spearman's correlation coefficients were calculated between each lectin (scGlycan-seq) and each of MYC (a), LEFTY1 (b), and LEFTY2 (c) genes (scRNA-seq) across hiPSCs (n = 42). Cells with a low number of detected genes were removed. * $p < 0.05$
